# Reverse Pathway Genetic Approach Identifies Epistasis in Autism Spectrum Disorders

**DOI:** 10.1371/journal.pgen.1006516

**Published:** 2017-01-11

**Authors:** Ileena Mitra, Alinoë Lavillaureix, Erika Yeh, Michela Traglia, Kathryn Tsang, Carrie E. Bearden, Katherine A. Rauen, Lauren A. Weiss

**Affiliations:** 1 Department of Psychiatry, University of California San Francisco, San Francisco, California, United States of America; 2 Institute for Human Genetics, University of California San Francisco, San Francisco, California, United States of America; 3 Université Paris Descartes, Sorbonne Paris Cité, Faculty of Medicine, Paris, France; 4 Department of Psychiatry and Biobehavioral Sciences, Semel Institute for Neuroscience and Human Behavior, University of California Los Angeles, Los Angeles, California, United States of America; 5 Department of Psychology, University of California Los Angeles, Los Angeles, California, United States of America; 6 Department of Pediatrics, School of Medicine, University of California San Francisco, San Francisco, California, United States of America; The Wellcome Trust Centre for Human Genetics, University of Oxford, UNITED KINGDOM

## Abstract

Although gene-gene interaction, or epistasis, plays a large role in complex traits in model organisms, genome-wide by genome-wide searches for two-way interaction have limited power in human studies. We thus used knowledge of a biological pathway in order to identify a contribution of epistasis to autism spectrum disorders (ASDs) in humans, a reverse-pathway genetic approach. Based on previous observation of increased ASD symptoms in Mendelian disorders of the Ras/MAPK pathway (RASopathies), we showed that common SNPs in RASopathy genes show enrichment for association signal in GWAS (*P* = 0.02). We then screened genome-wide for interactors with RASopathy gene SNPs and showed strong enrichment in ASD-affected individuals (*P* < 2.2 x 10^−16^), with a number of pairwise interactions meeting genome-wide criteria for significance. Finally, we utilized quantitative measures of ASD symptoms in RASopathy-affected individuals to perform modifier mapping via GWAS. One top region overlapped between these independent approaches, and we showed dysregulation of a gene in this region, *GPR141*, in a RASopathy neural cell line. We thus used orthogonal approaches to provide strong evidence for a contribution of epistasis to ASDs, confirm a role for the Ras/MAPK pathway in idiopathic ASDs, and to identify a convergent candidate gene that may interact with the Ras/MAPK pathway.

## Author Summary

The contribution of epistasis to human biology and complex trait architecture has been subject to intense debate. Despite statistical methods to detect interaction, allele frequencies and study designs have limited our power to address this question. Rather than a statistically-motivated approach, we developed a reverse pathway genetic approach to detect epistasis in in autism spectrum disorders (ASDs). Instead of traditional pathway analysis—exploiting unbiased genetic results to identify biological pathways important for pathophysiology—we hypothesized that the reverse approach of leveraging a specific biological pathway to uncover novel aspects of genetic architecture could be fruitful. We drew on knowledge from Mendelian disorders of the Ras/MAPK pathway (RASopathies) associated with ASD to inform our analyses. First, we implemented an epistasis screen in idiopathic ASD for SNPs that interact with the Ras/MAPK pathway, allowing us to detect genome-wide enrichment in epistasis signal as well as specific interacting loci. Second, we performed modifier mapping within RASopathies and identified overlapping signals with our first approach. Finally, we took the top overlapping region and experimentally demonstrated dysregulated expression in iPSC-derived neural cells from RASopathy subjects. Together, our results establish a human genetics motivated reverse-pathway strategy, which can be applied broadly to study epistasis.

## Introduction

### Common polymorphisms may contribute to neuropsychiatric disease beyond additive effects

Genome-wide association studies (GWAS) of common polymorphism association with complex neuropsychiatric traits have yielded recent success mapping single nucleotide risk polymorphisms with modest additive effects, particularly in schizophrenia[1]. However, complementary approaches utilizing the same data support an even greater role for common polymorphism in complex heritable disorders like schizophrenia and autism spectrum disorders (ASDs) than explained by the identified additive effects. For example, analysis in schizophrenia and other traits suggests that heritability is not completely accounted for by common single nucleotide polymorphisms (SNPs), in models inconsistent with contribution primarily from rare SNPs, suggesting that genetic interaction could account for some of the additional contribution of common variation[2,3]. Recent studies in ASDs estimate similarly large contribution of common variants to ASD liability despite inability to identify specific highly significant SNPs[4–6]. The modest effect sizes of individual SNP associations observed to date match expectation based on severe mating and fecundity reduction in schizophrenia and ASDs, however selective pressure could allow for stronger effects of gene-gene interaction, or epistasis[7,8].

### Epistasis is a pervasive genetic mechanism

Studies of complex traits in mice and in fruit flies have revealed modest main effects and frequency distributions similar to those identified in human GWAS[9]. However, careful study design and leverage of inbreeding have proven that epistasis typically accounts for a majority of the variation in quantitative traits[10–15]. Human GWAS studies have had limited success identifying genetic interactions, although model organism and theoretical evidence suggests that such effects are likely to be important[16]. Several limitations in human studies could account for this. First, power is extremely limited for genome-wide by genome-wide exploration of interaction, due to the potential number of even two-way tests to perform, requiring ‘astronomical’ sample sizes to even begin to address. Second, epistatic variance depends on both the size of genetic effects and the allele frequencies. Alleles with strong functional effects (such as those causing highly penetrant disease) may be more likely to show epistasis, but also be rare in the population. Therefore, in order to explore epistasis in humans, we wanted to take advantage of known rare, functional variation that can contribute to symptoms of complex disease[17].

### Mendelian disease genes contribute to common traits

Emerging evidence suggests that Mendelian diseases (high penetrance, dominant/recessive inheritance) resembling complex disorders may affect the same genes showing common risk variation in the population. There has been longstanding skepticism that similar phenotypes with simple and complex genetic inheritance share biological etiology, however, powerful recent study designs have revealed strong overlap. In Type II Diabetes, at least four of the first 20 genes shown robustly to be associated with the common adult-onset form were previously identified as causes of Mendelian forms[18]. Moreover, comprehensive analysis of medical records suggests widespread pleiotropy such that strong associations have been identified between single-gene Mendelian conditions and complex heritable traits; these specific associations are reflected in enrichment of GWAS SNP associations at the implicated Mendelian loci and increased replication at these loci compared with other GWAS-associated SNPs[19]. Further, this study indicated that these inferred genetic variants often act in a non-additive combinatorial model for certain disorders, including ASDs[19]. Despite strong evidence for association, many single-gene disorders display variation in penetrance or expression of associated complex phenotypes, i.e. reduced penetrance for these traits compared with primary features of Mendelian disease. One theory posits that background common genetic variation could modify risk for complex disease symptoms in the presence of a Mendelian disease. A notable example is autosomal recessive cystic fibrosis (CF), in which a combination of early gene identification (*CFTR*), common primary mutation (delta F508), frequency (1/3500 in the United States) and familial inheritance have enabled modifier mapping studies[20]. Many variable features of CF influence morbidity and mortality, including lung, liver, intestine, and pancreatic manifestations. Interestingly, in several examples so far, modifier loci in CF overlap with common complex traits[21]. *TGFB1* SNPs are associated with CF pulmonary function and with asthma and chronic obstructive pulmonary disease in the general population[22,23]. *TCF7L2*, *CDKAL1*, *CDKN2A/B*, and *IGF2BP2* and several other susceptibility loci for type 2 diabetes in the general population are also associated with high risk for CF-related diabetes[24]. Here, we can consider an independent SNP with large effect size in the presence of a Mendelian mutation (but modest effect size in the general population) to be equivalent to gene-gene interaction. One locus is known to be present due to affection with a monogenic disease, and the other is to be identified by modifier mapping. Thus, the active biological pathways involved in complex disease can be powerfully identified in studies with ascertainment for Mendelian conditions.

### ASDs are associated with Mendelian disorders of the Ras/MAPK pathway

ASDs are diagnosed based on core deficits in social reciprocity and communication as well as presence of restricted and repetitive behaviors, interests, or activities. These traits have also been long associated with a range of genetically simpler disorders, such as Fragile X syndrome, tuberous sclerosis, Rett syndrome, and Turner syndrome[25]. We hypothesized that Mendelian disorders associated with variable expression of ASD symptoms would be the optimal avenue for identification of gene-gene interaction. At the same time, specific study of natural variation in neurogenetic networks for behavioral traits in other organisms suggested a shift from considering single genes to pathway-based approaches[26]. Similarly, biological network knowledge has been proposed to enhance detection of epistasis[27–29]. We reasoned that a biologically informed network approach, showing promise in Crohn’s disease, bipolar disorder, hypertension and rheumatoid arthritis, may also illuminate ASD genetics[30,31]. Hence, instead of a single Mendelian disease, we chose to focus on a set of syndromes caused by mutations tightly intertwined in a single well-defined signaling pathway.

Disorders of the Ras/MAPK pathway (commonly referred to as RASopathies)[32] are ideal to study for identification of gene-gene interaction in ASD. Ras is a small GTPase with critical signaling functions in the cell, including the MAPK signaling cascade. Although best-known for its role in cancer due to acquired somatic mutations, dysregulation of genes in the Ras/MAPK pathway in human development causes disorders including neurofibromatosis type 1 (NF1: *NF1*[33]), Noonan syndrome and Noonan syndrome with multiple lentigines[34] (NS: *CBL*[35], *BRAF*, *KRAS*[36], *LZTR1*[37], *NRAS*[38,39], *PTPN11*[40], *RAF1*[41], *RASA2*[42], *RIT1*[43], *SHOC2*[44–46], *SOS1*[47,48], and *SOS2*[37]), Gingival fibromatosis 1 (*SOS1*[49,50]), Capillary malformation-arteriovenous malformation (CM-AVM) (*RASA1*[51,52]), Costello syndrome (CS: *HRAS*[53]), Cardio-facio-cutaneous syndrome (CFC[54]: *BRAF*,*MAP2K1*, *MAP2K2*, *KRAS*), and NF1-like syndrome (*SPRED1*[55]). Many of these syndromes share craniofacial dysmorphology, cardiac malformations and cutaneous, musculoskeletal and ocular abnormalities. We have recently studied four RASopathies (NF1, NS, CS, and CFC) and found association with both threshold measures correlated with clinical ASD diagnosis and quantitative ASD trait measures[56]. Our phenotype analyses suggested additional similarities with idiopathic ASDs, such as a male-biased sex ratio. Other groups have performed independent studies with highly consistent findings[57–62]. Studying multiple disorders in the same biological pathway could therefore both increase the power of our study and increase the likelihood of results translating to an even broader diagnostic category, idiopathic ASDs.

### Independent ‘reverse pathway analysis’ approaches

Instead of traditional pathway analysis—taking advantage of unbiased genetic analysis results to identify biological pathways important for disease pathophysiology—we hypothesized that the reverse approach of utilizing a specific biological pathway to uncover novel aspects of genetic architecture could be fruitful. In particular, we sought to overcome the limited power of genome-wide screens for two-way epistasis and the modest effects anticipated for common polymorphisms in complex disease. Thus, we designed two orthogonal approaches to leverage a well-defined biological pathway in order to learn more about gene-gene interaction in ASDs. 1) We performed a genetic screen for epistasis in idiopathic ASD subjects searching genome-wide for interaction partners of common polymorphisms in the Ras/MAPK pathway genes, thereby limiting one side of the pairwise test to include only polymorphisms relevant to a small number of RASopathy genes; 2) In parallel, we mapped SNPs influencing a quantitative measure of social responsiveness in RASopathy subjects ascertained for rare, major effect mutations in the Ras/MAPK pathway. In the latter case, the autosomal dominant RASopathy mutation is one locus, and the second locus involved in interaction will be identified by genome-wide SNP-based QTL mapping. We used these complementary approaches to show that ‘reverse pathway’ screening is a feasible approach to identify epistasis relevant to a complex heritable trait.

## Results

### Main effects of Ras/MAPK polymorphisms in ASDs

**Fig 1:**
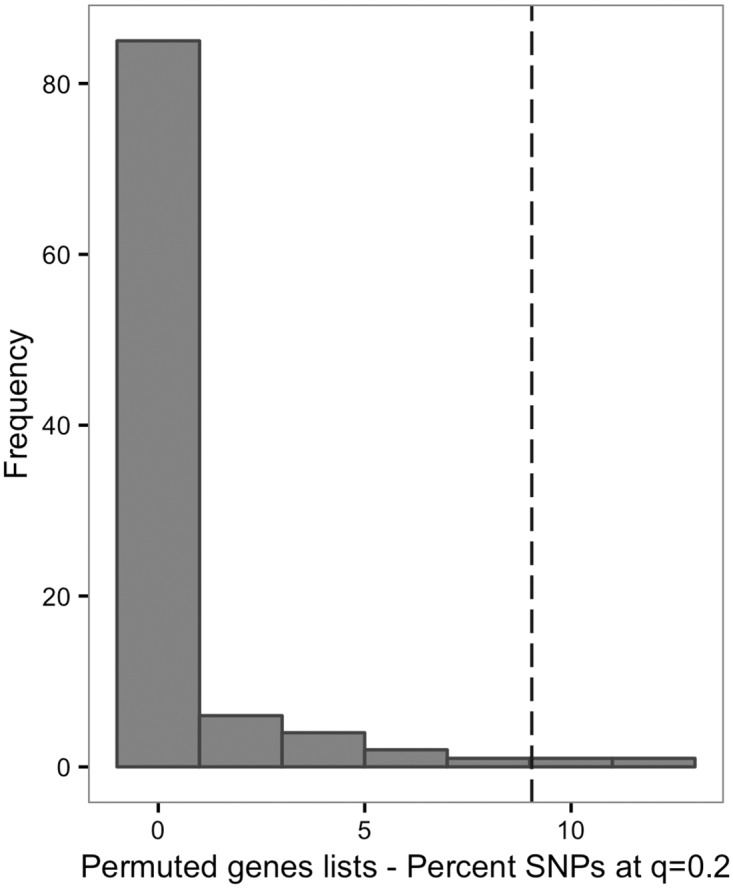
Enrichment of Ras/MAPK SNPs in ASD association results. The histogram displays the distribution of results with percent of SNPs meeting q = 0.2 for 100 randomly permuted gene sets compared to the Ras/MAPK SNP set (dashed line, 9.05%) in an ASD transmission disequilibrium test (TDT) for association. The y-axis displays the proportion of permutation results; the x-axis displays the percent SNPs meeting q = 0.2.

We generated an idiopathic ASD GWAS dataset by utilizing each available published dataset [Autism Genetic Resource Exchange (AGRE), Autism Genome Project (AGP), Simons Simplex Collection (SSC)] in addition to in-house generated data [University of California, San Francisco (UCSF)], all of which were comprised of family trios with one affected offspring and both parents. We performed a transmission disequilibrium test (TDT) for ASD association in our final dataset of 4,109 trios. We first performed set-based analysis for polymorphisms within 5kb of each of the known RASopathy genes in the Ras/MAPK pathway (S1 Table). Then, we compared the proportion of SNPs exceeding a false discovery rate (FDR) threshold of q = 0.2 to permutations using length-matched random gene sets, showing that common polymorphisms within Ras/MAPK genes are significantly enriched for association with idiopathic ASDs (*P* = 0.02) (Fig 1). This evidence for main effects of Ras/MAPK polymorphism in ASDs supports our rationale for testing epistasis involving Ras/MAPK SNPs.

### Epistasis with Ras/MAPK polymorphisms in ASD subjects

**Fig 2:**
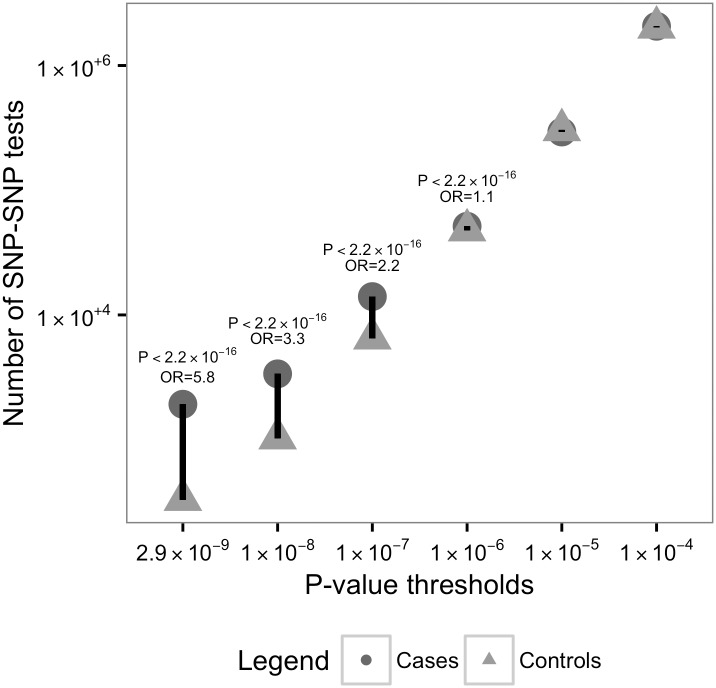
Comparison of number of Ras/MAPK gene epistasis results in ASD cases versus pseudo-controls. The graph displays number of epistasis tests (y-axis) in the ASD cases (dark gray, circle) and ASD pseudo-controls (light gray, triangle) with *P*-value thresholds (x-axis, left to right): *P* < 2.9x10^-9^, < 1.0x10^-8^, *P* < 1.0x10^-7^, *P* < 1.0x10^-6^, *P* < 1.0x10^-5^, and *P* < 1.0x10^-4^. The 2x2 chi-square test *P*-value and odds ratio (OR) are included for the epistasis results meeting nominal significance (*P* < 10^−6^).

We next performed ‘set-by-all’ tests for two-way genetic interactions with polymorphisms in the Ras/MAPK SNP set (S1 Table). This is performed by testing each polymorphism in the Ras/MAPK SNP set (SNP 1) for correlation with any other polymorphism (on an unlinked autosome) across the genome (SNP 2) in cases with ASDs. SNP1-SNP2 correlation across independent chromosomes in a genetically homogeneous population is considered evidence for epistasis contributing to disease risk. We calculated a genome-wide significance threshold (*P* = 7.6x10^-10^) accounting for multiple LD groups per Ras/MAPK gene and the proportion of the genome tested for each genome-wide epistasis screen (see S2 Table). At this threshold, 569 SNP pairs representing 19 independent region pairs showed epistasis in cases, which was significantly increased compared with matched pseudo-controls (OR 3.1, *P* < 2.2x10^-16^). We observed an excess of association at several additional *P*-value thresholds in this analysis in ASD cases compared with matched pseudo-controls (*P* < 2.2x10^-16^) (Table 1, Fig 2). We also identified individual epistasis signals surpassing a gene-based significance threshold set by dividing the GWAS significance threshold (*P* = 5.0x10^-8^) by the number of genes in the Ras/MAPK set for an approximate independent hypothesis-testing estimate (*P* = 2.9x10^-9^) considering all SNPs in a single gene part of the same hypothesis (i.e. LD-independent SNPs relevant to the same gene considered non-biologically independent due to potentially similar functional consequences) (Table 1, S1A–S1F Fig). The data underlying these results show that the primary driving genotype-combination category is double-heterozygotes, which shows dramatic increase compared with expected counts in cases, but similar to expected counts in pseudo-controls (S3 Table). This would be expected for minor allele interactions increasing risk, as most combinations including minor allele homozygotes have very low counts.

**Table 1:** Ras/MAPK ASD epistasis top results. The unique epistatic SNP pairs with *P* < 2.9x10^-9^ are listed in the table. For each SNP, the following is listed in order of columns: rsID (Epistatic SNP), chromosome (CHR), position (BP, reference version hg19), minor allele frequency in the ASD dataset (MAF), nearest gene to the epistatic SNP, Ras/MAPK gene associated with the interacting SNP, and *P*-value for epistasis in cases (Epistasis ASD *P*) and pseudo-controls (Epistasis Control *P*). Locus pairs meeting genome-wide significance criteria (*P* < 7.6 x 10^−10^) are bolded. Main effects for epistatic and Ras/MAPK SNPs listed here are listed in S4 Table, with no SNPs showing *P* < 0.01.

Epistatic SNP	CHR	BP	MAF	Nearest gene (epistatic SNP)	Ras/ MAPK pathway gene	Epistasis ASD *P*	Epistasis control *P*
**rs149565205**	**3**	**6,539,899**	**0.03**	***EDEM1 / GRM7***	***PTPN11***	**7.3x10**^**-11**^	**0.018**
**rs114490548**	**7**	**3,8139,570**	**0.02**	***EPDR1 / STARD3NL***	***RASA1***	**7.8x10**^**-11**^	**0.39**
**rs2441690**	**11**	**127,107,790**	**0.06**	***KIRREL3 / ETS1***	***MAPK1***	**1.0x10**^**-10**^	**0.17**
**rs62168052**	**2**	**135,088,091**	**0.03**	***MGAT5***	***SPRED1***	**1.1x10**^**-10**^	**0.21**
**rs304654**	**4**	**124,123,184**	**0.02**	***SPATA5***	***SOS1***	**1.1x10**^**-10**^	**0.93**
**rs56667163**	**3**	**136,773,620**	**0.06**	***IL20RB / SOX14***	***RASA1***	**1.6x10**^**-10**^	**0.73**
**chr15:71697182:D**	**15**	**71,697,182**	**0.02**	***THSD4***	***RAF1***	**1.6x10**^**-10**^	**0.25**
**rs78762238**	**13**	**64,302,181**	**0.03**	***PCDH20/ PCDH9***	***NF1***	**2.5x10**^**-10**^	**9.4x10**^**-3**^
**rs41274082**	**10**	**65,381,281**	**0.02**	***REEP3***	***MAP2K1***	**2.6x10**^**-10**^	**0.70**
**rs60709797**	**15**	**23,004,989**	**0.03**	***NIPA2***	***PTPN11***	**2.7x10**^**-10**^	**9.9x10**^**-3**^
**rs11925140**	**3**	**112,842,425**	**0.02**	***GTPBP8 / BOC***	***NF1***	**3.7x10**^**-10**^	**0.05**
**rs57173428**	**1**	**55,100,883**	**0.02**	***ACOT11 / TTC4***	***KRAS***	**3.8x10**^**-10**^	**0.25**
**rs1826547**	**8**	**27,414,024**	**0.03**	***EPHX2 / SCARA3***	***BRAF***	**4.0x10**^**-10**^	**0.48**
**rs17110869**	**1**	**55,108,237**	**0.02**	***MROH7***	***KRAS***	**4.5x10**^**-10**^	**0.26**
**rs80179511**	**6**	**154,274,998**	**0.06**	***RGS17 / OPRM1***	***RAF1***	**5.3x10**^**-10**^	**0.16**
**rs140695911**	**10**	**29,495,250**	**0.02**	***BAMBI / LYZL1***	***KRAS***	**5.9x10**^**-10**^	**0.29**
**rs28459694**	**1**	**158,355,190**	**0.03**	***CD1E / OR10T2***	***MAPK1***	**6.2x10**^**-10**^	**0.28**
**rs73760016**	**5**	**19,469,048**	**0.03**	***BASP1 / CDH18***	***SPRED1***	**6.8x10**^**-10**^	**0.036**
**rs192196641**	**10**	**8,587,332**	**0.04**	***GATA3 / CELF2***	***MAP2K1***	**6.8x10**^**-10**^	**0.062**
rs80214471	5	66,139,877	0.03	*MAST4*	*MAP2K1*	8.4x10^-10^	0.60
rs2765709	10	25,507,753	0.10	*GPR158*	*MAPK1*	9.0x10^-10^	0.39
rs2043732	7	51,192,161	0.06	*COBL*	*RASA2*	1.3x10^-9^	0.050
rs114617777	6	88,085,754	0.06	*SMIM8 / CFAP206*	*SOS1*	1.3x10^-9^	0.19
chr13:78700408:D	13	78,700,408	0.02	*EDNRB / POU4F1*	*SPRED1*	1.4x10^-9^	0.46
rs72690923	1	106,012,196	0.03	*AMY1B / PRMT6*	*BRAF*	1.4x10^-9^	0.66
rs4128728	18	49,575,401	0.03	*MEX3C / DCC*	*SOS1*	1.6x10^-9^	0.17
rs11255742	10	8,598,746	0.03	*KRT8P16*	*MAP2K1*	1.6x10^-9^	0.087
chr6:80625529:D	6	80,625,529	0.13	*ELOVL4*	*SHOC2*	1.7x10^-9^	0.89
rs73688732	7	31,068,898	0.02	*GHRHR / ADCYAP1R1*	*MAP2K1*	1.9x10^-9^	0.35
rs1318299	2	220,283,259	0.02	*DES*	*BRAF*	2.1x10^-9^	0.47
chr12:21624694:D	12	21,624,694	0.10	*RECQL*	*SOS1*	2.2x10^-9^	0.81
rs73475884	13	35,080,882	0.07	*RFC3 / NBEA*	*SHOC2*	2.2x10^-9^	0.28
rs58413939	15	23,003,403	0.04	*CYFIP1*	*RASA1*	2.3x10^-9^	0.031
rs143823697	5	134,412,896	0.03	*PITX1 / H2AFY*	*MAPK1*	2.4x10^-9^	0.40
rs12239450	1	47,933,492	0.09	*FOXD2 / TRABD2B*	*BRAF*	2.4x10^-9^	0.58
rs113552799	6	123,947,624	0.04	*TRDN*	*MAP2K1*	2.5x10^-9^	0.80
rs254700	5	134,103,554	0.02	*DDX46*	*SHOC2*	2.6x10^-9^	0.37
rs12582581	12	511,258	0.02	*CCDC77*	*CBL*	2.6x10^-9^	0.26
rs55942942	13	35,095,658	0.07	*RFC3*	*SHOC2*	2.7x10^-9^	0.29
rs118078508	6	154,999,201	0.02	*CNKSR3 / SCAF8*	*PTPN11*	2.8x10^-9^	0.44

We performed a number of negative and positive control analyses in order to exclude any bias or artifact. For our first negative control, we performed similar set-by-all epistasis testing for a permuted SNP set (median result in the main effect enrichment analysis above). In testing for epistasis enrichment for a non-candidate pathway-selected set, we do not observe enrichment of epistasis results in cases compared to pseudo-controls at our top significance thresholds (OR < 1) (S2 Fig). Second, for our observed Ras/MAPK results above, we not only compared a pseudo-control correlation approach (as above; in Table 1 all 41 top SNP pairs show different OR between cases and pseudo-controls *P* < 0.05), but also used matched-sex parent controls, which provided similar results to pseudo-controls (no epistasis *P* < 10^−6^ for SNP pairs in Table 1; significant overrepresentation genome-wide in cases at all *P*-value thresholds considered from *P* < 10^−6^ to *P* < 7.6 10^−10^). As a positive control, we selected the small homogeneous set of cases and matched unaffected siblings from unique families (N = 1,136) from the SSC dataset, and similarly to pseudo-controls, we observed significant epistasis enrichment in cases and diminishing odds ratios at varying *P*-value thresholds down to *P* < 10^−6^ (S3 Fig). Finally, we validated the top results (*P* < 1.0x10^-6^) with the trio correlation test for epistasis. This independent method had highly correlated *P*-values with the case-only approach, suggesting the results are consistent with epistasis and not marginal effects (rho = 0.78, *P*< 2.2x10^-16^). We observed that all results from the PLINK[63,64] case-only epistasis analysis with *P*< 1.0x10^-6^ also had *P*< 4.4x10^-5^ in the trio correlation test, thereby confirming the results with an independent method (S4 Fig).

### Mapping of an ASD-related quantitative trait in RASopathy subjects

**Fig 3:**
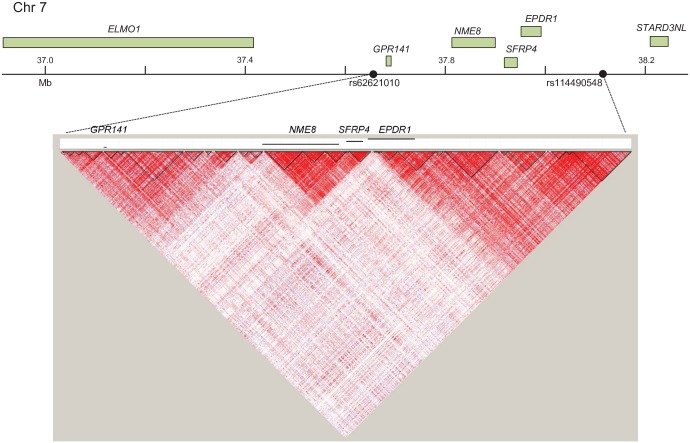
Linkage disequilibrium (LD) map of the region chromosome 7: 37.7Mb– 38.1 Mb. The graph displays LD between the SNPs rs114490548 (*P* = 7.8 x10^-11^, Ras/MAPK ASD epistasis analysis) and rs62621010 (*P* = 5.6x10^-7^, RASopathy QTL analysis). LD (D′) values for each pairwise comparison of SNPs were calculated based on LD and recombination rate data in 1000 Genomes May 2013 release and plotted using HAPLOVIEW(126) (see web resources) default settings and standard color theme. The red color corresponds to D’ = 1 and log of odds (LOD) ≥ 2, white corresponds to D’<1 and LOD <2, and blue to D’ = 1 and LOD<2.

In order to utilize a separate approach to assess evidence for multiple genetic hits contributing to ASD symptoms, we ascertained individuals for having a RASopathy (NF1, NS, CS, and CFC). Although these individuals have a dominant germline mutation in the Ras/MAPK pathway causing some highly penetrant features, we have previously shown that ASD symptoms are variable both across and within disorders[56]. Thus, we performed a modifier screen via genome-wide association based on a quantitative social responsiveness trait, measured by the Social Responsiveness Scale (SRS) (S5 Fig). The SRS is a questionnaire measure with normally distributed and highly heritable scores in the general population strongly reflective of clinical ASDs[65]. Thus, identifying modifiers of an ASD-related trait in individuals ascertained for a RASopathy could identify interactors with the Ras/MAPK pathway relevant to ASDs, as we consider the RASopathy locus and the modifier locus interacting to influence the ASD-relevant trait. We performed quantitative trait locus (QTL) mapping within-disorder and meta-analysis across the four disorders, as well as QTL mapping in sibling controls. We did not observe any SNPs meeting criteria for genome-wide significance (*P* = 5.0x10^-8^), however, the most significant SNP in this QTL analysis (rs62621010, *P* = 5.6x10^-7^, Table 2) is 0.39 Mb from the locus with the second-most significant epistasis signal in ASDs (rs114490548, Table 1). The rs62621010 putative modifier SNP does not show evidence for association with SRS in sibling controls. The region flanking and between rs62621010 and rs114490548 (chr7:37,749,392–38,139,570) contains genes *ELMO1*, *GPR141*, *NME8*, *SFRP4*, *EPDR1*, and *STARD3NL*. The two SNPs have low LD in 1000genomes measured by *r*^*2*^, although there is high D’ variably across the region (Fig 3). Neither SNP is represented in both ASD and RASopathy datasets (or can be imputed) for direct comparison.

**Table 2:** Social responsiveness association in RASopathy top results. The independent SNPs with social responsiveness score (SRS) association in RASopathy (random effects meta-analysis *P* < 1.0x10^-4^) are listed. The data underlying the top six candidate modifiers are graphically illustrated in S6 Fig. For each SNP, the following is listed in order of columns: SNP rsID, chromosome (CHR), position (BP, reference version hg19), minor allele frequency in the dataset (MAF), groups contributing to the RASopathy association (group with the most significant association *P*-value is listed first and groups with similar direction of effect are in parentheses), Cochran’s Q *P*-value for all four RASopathy groups, RASopathy (CFC, CS, NF1, and NS) SRS association (random effects meta-analysis) *P*-value, control sibling SRS *P*-value (linear regression), gene(s) containing or flanking SNP.

SNP	CHR	BP	MAF	RASopathy	RASopathy heterogeneity *P*	RASopathy *P*	Sibling *P*	Nearest gene(s)
rs62621010	7	37,749,392	0.02	NF1 (CS)	3.7x10^-7^	5.6x10^-7^	0.87	*ELMO1 / GPR141*
rs531418	5	163,079,682	0.08	NF1 (CFC, CS, NS)	0.82	1.4x10^-6^	0.016	*MAT2B*
rs11013152	10	23,137,611	0.26	CS (CFC, NF1)	1.0x10^-4^	1.8x10^-6^	0.82	*PIP4K2A*
rs117802216	11	25,911,732	0.05	NF1 (CFC, CS, NS)	0.057	2.0x10^-6^	0.33	*LUZP2*
rs1028948	20	16,213,705	0.12	CFC (CFC, CS, NF1, NS)	0.66	2.1x10^-6^	0.84	*MACROD2*
rs149068014	11	118,639,179	0.01	NF1 (CS, NF1)	1.8x10^-4^	2.2x10^-6^	0.37	*DDX6*
rs6518584	22	19,821,819	0.44	CS (CFC, NF1, NS)	0.061	2.5x10^-6^	0.36	*GNB1L*
rs11573662	9	110,063,197	0.14	CS (CFC, NF1, NS)	0.53	3.3x10^-6^	0.66	*RAD23B*
rs117695738	15	100,630,815	0.05	CS (CFC, NF1, NS)	0.30	4.6x10^-6^	0.54	*ADAMTS17*
rs646770	13	103,325,307	0.33	NF1 (CFC, CS, NS)	0.076	4.8x10^-6^	0.92	*TPP2*
rs486955	10	104,546,284	0.13	NS (CFC, CS, NF1)	0.14	5.4x10^-6^	0.72	*WBP1L*
rs12594951	15	42,934,631	0.46	CS (CFC, NF1, NS)	0.071	5.9x10^-6^	0.21	*STARD9*
rs2139219	4	165,979,548	0.04	NF1 (CFC, CS, NS)	0.017	6.0x10^-6^	0.067	*TMEM192*
rs4680721	3	29,413,623	0.12	CFC (CS, NF1, NS)	0.32	7.3x10^-6^	0.16	*RBMS3*
rs12209962	6	6,451,710	0.41	CS (NF1, NS)	3.3x10^-5^	1.2x10^-5^	0.26	*LY86*
rs12518526	5	77,621,049	0.49	CFC (CS, NF1, NS)	0.42	1.3x10^-5^	0.51	*AP3B1 / SCAMP1*
rs59627556	16	82,130,477	0.08	NF1 (CFC, NS)	0.031	1.3x10^-5^	0.88	*HSD17B2*
rs57626469	16	12,577,817	0.10	CS (CFC, NF1, NS)	0.041	1.3x10^-5^	0.50	*SNX29*
rs80270452	1	205,539,721	0.01	NF1 (CFC)	0.033	1.3x10^-5^	0.058	*MFSD4*
rs9518224	13	101,597,633	0.36	CFC	2.2x10^-6^	1.6x10^-5^	0.66	*NALCN*
rs7962094	12	109,008,869	0.30	CS (CFC, NF1, NS)	0.22	1.7x10^-5^	0.21	*SELPLG*
rs77503217	11	127,817,436	0.05	NF1 (CS, NS)	3.7x10^-5^	2.0x10^-5^	0.19	*KIRREL3 / ETS1*
rs75450294	11	97,570,766	0.03	CFC (NF1, NS)	0.39	2.0x10^-5^	0.74	*CNTN5*
rs112114585	12	119,209,797	0.04	NF1 (CFC, CS, NS)	3.4x10^-3^	2.1x10^-5^	0.90	*SUDS3*
rs75553973	7	42,656,800	0.02	NF1 (CS, NS)	0.95	2.1x10^-5^	0.71	*GLI3*
rs62221469	21	46,282,961	0.25	CS (CFC, NF1)	0.054	2.2x10^-5^	0.50	*PTTG1IP*
rs73141393	12	70,832,093	0.06	NS (CFC, CS, NF1)	0.39	2.3x10^-5^	0.76	*KCNMB4*
rs6075577	20	19,809,524	0.47	CFC (CS, NF1, NS)	0.24	2.5x10^-5^	0.68	*SLC24A3 / RIN2*
rs117592064	10	90,787,531	0.02	CFC (CS, NF1, NS)	0.59	2.6x10^-5^	0.17	*FAS*
rs79497225	12	19,703,676	0.02	NF1 (CFC)	0.022	2.8x10^-5^	0.89	*AEBP2*
rs11689566	2	118,966,616	0.06	NF1 (CFC, CS, NS)	0.26	2.8x10^-5^	0.34	*INSIG2 / EN1*
rs10463788	5	124,564,573	0.25	NF1 (CFC, CS, NS)	0.47	2.8x10^-5^	0.71	*ZNF608 / GRAMD3*
rs13026531	2	137,805,397	0.18	NS (CS)	1.5x10^-6^	3.0x10^-5^	0.10	*THSD7B*
rs6516036	20	5,422,539	0.19	CFC (CFC, CS, NF1, NS)	0.51	3.1x10^-5^	0.78	*PROKR2 / GPCPD1*
rs11106988	12	93,734,450	0.47	NS (CFC, CS, NF1)	0.37	3.1x10^-5^	0.73	*EEA1 / NUDT4*
rs11527676	10	36,333,349	0.39	NS (CFC, CS, NF1)	0.41	3.2x10^-5^	0.48	*FZD8 / ANKRD30A*
rs2874027	5	24,485,005	0.13	CFC (CS, NF1, NS)	0.35	3.3x10^-5^	0.38	*CDH10*
rs17634284	9	122,091,015	0.08	NF1 (CFC, CS, NS)	0.88	3.3x10^-5^	0.24	*BRINP1*
rs36081923	20	9,955,080	0.21	CS (CFC, NF1, NS)	0.91	3.4x10^-5^	0.82	*PAK7 / ANKEF1*
rs116261553	2	28,606,305	0.02	NF1 (CFC, NS)	0.031	3.6x10^-5^	0.77	*BRE / FOSL2*
rs1990743	17	63,943,956	0.44	NF1 (CFC, CS, NS)	0.14	3.7x10^-5^	0.35	*CEP112*
rs12827688	12	56,309,504	0.08	NF1 (CFC, CS, NS)	0.012	3.9x10^-5^	0.81	*WIBG*
rs496957	15	46,875,541	0.19	CFC (CFC, CS, NS)	0.47	4.0x10^-5^	0.61	*SQRDL / SEMA6D*
rs79448371	5	80,709,755	0.02	NF1 (CFC, CS, NS)	0.92	4.0x10^-5^	0.55	*ZCCHC9 / SSBP2*
rs6510032	19	57,487,513	0.09	NF1 (CS)	3.7x10^-3^	4.3x10^-5^	0.37	*USP29*
rs3807822	7	8,247,347	0.06	NF1 (CFC, NS)	0.059	4.3x10^-5^	0.02	*ICA1*
rs17834272	10	108,046,638	0.30	CS (CFC, NF1, NS)	0.47	4.5x10^-5^	0.82	*SORCS3 / SORCS1*
rs9519852	13	106,574,453	0.21	CS (CFC, NF1, NS)	0.85	4.7x10^-5^	0.76	*DAOA / EFNB2*
rs78471416	2	105,907,528	0.04	NF1 (CS, NS)	0.045	4.7x10^-5^	0.79	*TGFBRAP1*
rs10060705	5	63,901,304	0.49	NS (CFC, CS, NF1, NS)	0.23	4.7x10^-5^	0.73	*RGS7BP*
rs545076	12	115,119,903	0.50	CS (CFC, NF1, NS)	0.38	4.9x10^-5^	0.89	*TBX3*
rs12634256	3	160,907,163	0.18	NF1 (CFC, CS, NS)	0.95	5.0x10^-5^	0.47	*B3GALNT1 / NMD3*
rs62323999	4	162,217,243	0.03	NS (CFC, CS, NF1)	0.76	5.3x10^-5^	0.43	*RAPGEF2 / FSTL5*
rs117872061	7	82,125,674	0.01	NF1 (CFC, CS, NS)	0.54	5.4x10^-5^	0.38	*CACNA2D1 / PCLO*
rs76013032	1	108,421,826	0.04	CFC (CS, NF1, NS)	0.27	5.5x10^-5^	0.67	*VAV3*
rs7445190	5	63,911,811	0.28	NS (CS, NF1)	0.042	5.5x10^-5^	0.65	*RNF180 / FAM159B*
rs4924250	15	38,675,052	0.09	NF1 (CS)	9.6x10^-5^	6.2x10^-5^	0.50	*SPRED1 / FAM98B*
rs62245932	3	68,177,506	0.09	CFC (CS, NF1, NS)	0.51	6.2x10^-5^	0.65	*FAM19A1*
rs13112607	4	30,770,684	0.04	NF1 (CFC, CS, NS)	0.91	6.6x10^-5^	0.032	*PCDH7*
rs881122	11	117,972,936	0.23	NF1 (CFC, CS, NS)	0.52	6.7x10^-5^	0.15	*TMPRSS4*
rs35206247	16	7,112,749	0.13	NF1 (CFC, CS, NS)	0.027	6.8x10^-5^	0.44	*RBFOX1*
rs117101324	14	76,851,865	0.04	NF1 (CFC, CS, NS)	0.013	6.8x10^-5^	0.58	*ESRRB*
rs2064049	21	16,938,815	0.18	NS (CFC, CS, NF1)	0.90	7.0x10^-5^	0.41	*NRIP1 / USP25*
rs72695575	4	167,648,687	0.04	CFC (CS, NF1)	0.15	7.2x10^-5^	0.18	*TLL1 / SPOCK3*
rs1477910	8	89,093,270	0.04	NF1 (CS, NS)	0.037	7.5x10^-5^	0.65	*MMP16*
rs11108403	12	96,483,815	0.15	CS (CFC, NF1, NS)	0.063	7.5x10^-5^	0.089	*LTA4H / ELK3*
rs78665078	4	47,060,825	0.03	NF1 (CFC, NF1)	3.8x10^-4^	7.6x10^-5^	0.85	*GABRB1*
rs12746755	1	232,014,313	0.01	CS (CFC, NF1, NS)	0.56	7.8x10^-5^	0.72	*DISC1*
rs17034964	4	157,531,487	0.31	CS (CFC, NF1, NS)	0.13	7.8x10^-5^	0.082	*CTSO / PDGFC*
rs75082290	2	67,831,153	0.10	CS (CFC, NF1, NS)	0.25	7.9x10^-5^	0.037	*ETAA1 / C1D*
rs73879235	3	170,626,704	0.03	NF1 (CFC, CS, NS)	0.81	8.1x10^-5^	0.96	*EIF5A2*
rs2762659	10	10,164,309	0.25	CFC (CS, NF1, NS)	0.91	8.2x10^-5^	0.49	*GATA3 / CELF2*
rs514614	18	362,173	0.50	NF1 (CFC, CS, NS)	0.56	8.2x10^-5^	0.24	*COLEC12*
rs73209085	3	194,253,965	0.04	NS (CS, NF1)	0.96	8.4x10^-5^	0.20	*U6*
rs73008243	6	144,394,296	0.08	NF1 (CS)	1.0x10^-4^	8.8x10^-5^	0.55	*PLAGL1/ SF3B5*
rs79877255	18	62,662,276	0.02	NF1 (CFC, NS)	0.24	9.0x10^-5^	0.84	*SERPINB8 / CDH7*
rs17728300	3	8,600,038	0.02	NF1 (NS)	3.8x10^-5^	9.1x10^-5^	0.76	*LMCD1*
rs16875506	5	77,788,791	0.34	NS (CFC, CS, NF1)	0.95	9.6x10^-5^	0.72	*LHFPL2*
rs2142967	1	209,711,015	0.06	CFC (CS, NF1, NS)	0.31	9.6x10^-5^	6.4x10^-3^	*CAMK1G / LAMB3*
rs3732209	2	128,079,806	0.31	NS (NF1)	6.6x10^-4^	9.8x10^-5^	0.18	*MAP3K2*
rs77320284	10	109,037,309	0.03	NF1 (NS)	7.2x10^-4^	1.0x10^-4^	0.27	*SORCS1 / XPNPEP1*
rs79534834	3	119,267,493	0.16	NS (NF1)	1.8x10^-5^	1.0x10^-4^	0.85	*CD80*

### Expression dysregulation in RASopathy neural cell lines

**Fig 4:**
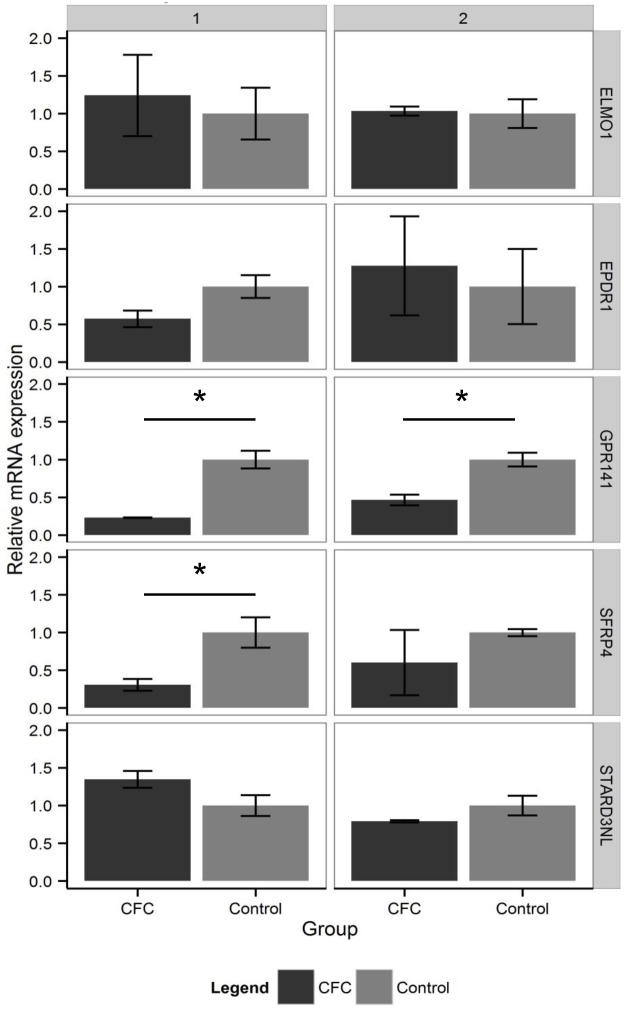
Gene expression in neural cell lines. For the genes *ELMO1*, *GPR141*, *SFRP4*, *EPDR1*, *and STARD3NL*, the graph displays the normalized mRNA expression relative to controls measured by qPCR for two independent experiments. *NME8* had undetermined quantities in the first experiment (1) and extreme variance in the second experiment (2) due to low expression level, and therefore was excluded from the graph (S3 Table). The error bars denote the standard error of the sample measurements, and the asterisk denotes a t-test *P*-value < 0.05 between CFC (dark gray) and control (light gray).

For prioritization of genes within the top region jointly identified by epistasis analysis in idiopathic ASDs and QTL-mapping in RASopathy subjects, we assessed expression level of genes in the chromosome 7 region in RASopathy neural cell lines. Our reasoning was that if a RASopathy mutation resulted in expression dysregulation of a gene, it provides strong biological plausibility for interaction with Ras/MAPK signaling. We utilized qRT-PCR to compare RNA extracted from CFC (*BRAF* c.770A>G, p.Q257R) patient-derived iPSC neural cell cultures (5 weeks) and compared to control-derived matched cultures[66]. All cultures were positive for mature neuronal and astrocyte markers, *MAP2* and *GFAP* (S7 Fig). In the first experiment (CFC N = 3; control N = 3; performed in technical triplicates), we identified two genes appearing to be downregulated in CFC lines (*GPR141*, *P* = 0.02; *SFRP4*, *P* = 0.03, S5 Table, Fig 4). In order to independently replicate this result, we utilized RNA extracted from a second batch of independently-derived neural lines from a set of overlapping individuals at 5 weeks (CFC N = 3; control N = 2; performed in technical triplicates), and showed that *GPR141* was again significantly downregulated (*P* = 0.04, S5 Table, Fig 4). *GPR141* encodes a brain-expressed orphan g-protein coupled receptor in the rhodopsin family.

## Discussion

We find the results of this reverse-pathway genetic study compelling for a number of reasons. We limit one side of a two-way interaction test to a relevant genetic pathway, and obtain genome-wide enrichment for epistasis in a common, complex human disease. Further, specific epistasis results survive independent genome-wide or gene-by-genome multiple testing correction, and reside near compelling candidate loci. Our second approach of modifier mapping for a relevant quantitative trait in subjects with Mendelian disorders in the same pathway, RASopathies, converges on several regions overlapping the ASD epistasis results. A gene in the top overlapping region across analyses showed expression dysregulation in neural cell lines from RASopathy subjects. Together, our results from each step of this study suggest we have identified powerful approaches to unravel complex genetic mechanisms.

First, in a reverse pathway-driven approach, we obtained strong evidence for gene-gene interaction in a human complex trait. We have previously used candidate-pair approaches to find interaction effects in ASDs, which have been independently replicated and shown to have a functional basis[67–71], and other studies have performed epistasis screens limiting all discovery to a candidate set or pathway[72–76]. However, our set-by-genome strategy can not only identify novel disease-relevant biological relationships, but this study also directly shows a genome-wide excess of epistasis signal in human disease (*P* < 2.2 x 10^−16^), achieved by constraining the set of interaction partners based on biological and genetic knowledge. The approach we have used here is in principle straightforward and could be applied to other disorders or pathways.

Beyond global enrichment for epistasis in ASDs, we identified specific loci meeting criteria for gene-based or genome-wide significance considering the number of Ras/MAPK genes or LD groups for which genome-wide screening was performed (*P* < 2.9 x 10^−9^; *P* < 7.6 x 10^−10^). Loci identified to be putative Ras/MAPK interaction partners influencing ASD risk in this way include or are adjacent to some already strong ASD candidate genes. *GRM7* has been identified in rare, *de novo* CNV deletions[77,78] and single nucleotide mutations[79] in subjects with ASD and in a candidate gene SNP study. It encodes a metabotropic glutamate receptor critical for early development[80]. *KIRREL3* has likewise been implicated by rare genetic disruptions[81–84] and inclusion in expression networks of common polymorphism association[85], as well as mouse behavioral anomalies[86]. It is thought to be key for synaptogenesis. *NIPA2* and *CYFIP1* are located on 15q11.2 in the region affected by an interstitial microduplication syndrome associated with ASDs[87,88]. *PCDH9*, *PITX*, *REEP3*, *NBEA*, and *OPRM1* are additional ASD candidate genes listed in SFARIgene[89] (see web resources).

Similarly, several genes implicated by this epistasis analysis are specifically relevant to the Ras/MAPK pathway. *SPRY1* encodes a classic inhibitor of the Ras/MAPK pathway, and plays a critical role in determining the balance between proliferation and differentiation for cortical patterning and cerebellar development[90–92]. *DCC* encodes the receptor deleted in colorectal cancer (DCC), which recruits proteins to promote axon outgrowth and guidance during neurodevelopment, and has been shown to interact with a Ras inhibitor[93]. *GATA3* is a transcription factor known for its role in T-cell development, however it also helps to control excitatory/inhibitory balance by determining GABAergic vs. glutamatergic fates during neurodevelopment[94]. Ras/MAPK signaling can regulate the stability of GATA3 post-transcriptionally by inhibiting the ubiquitin-proteasome pathway[95]. *GHRHR* encodes the growth hormone releasing hormone receptor which can activate Ras/MAPK signaling[96].

Second, our results are in line with the previous prediction that not only would non-additive effects be a major contributor to ASDs, but also that Mendelian syndromic genes are likely to be enriched for main effects[19]. We observe enrichment of additive association signal for common polymorphisms near the Ras/MAPK genes, supporting traditional pathway analyses that have identified this pathway as a major contributor to ASDs and indicating general overlap in biological etiology between Mendelian and complex traits [97–99]. In addition, our results in idiopathic ASD SNP datasets strongly support our findings in rare RASopathies. One of the highly significant epistasis loci is near the top result (albeit non genome-wide significant) in a QTL modifier mapping approach in RASopathy subjects. A gene in this region, *GPR141*, demonstrates reproducibly reduced expression in RASopathy neural cell lines. Although this is merely circumstantial with regards to the observed epistasis and QTL results, it provides biological plausibility for interaction with the Ras/MAPK pathway.

The function of GPR141, an orphan g-coupled protein receptor, is currently unknown. Sequence-structure based phylogeny suggests potential ligand association with N-arachidonylglycine (NAGly)[100]. The well-studied GPR18 in this class has been shown to mediate concentration-dependent phosphorylation of ERK 1/2 in the presence of NAGly[101]. NAGly is known to be anti-nociceptive and is thought to reversibly inhibit calcium currents in sensory neurons and have additional minor effects on sodium currents[102]. Further experimentation specific to GPR141 would be necessary to speculate about its potential interaction with the Ras/MAPK pathway or functional role in ASDs. A second gene in the region, *SFRP4*, showed nominal reduced expression in our first experiment and non-significant but consistent expression reduction in the second experiment. Secreted frizzled-related protein 4 (SFRP4) is an antagonist for Wnt ligands, inhibiting the canonical Wnt signaling pathway. Based on both linkage disequilibrium in the region and our experimental results, we cannot rule out that there are two separate loci or genes of interest relevant to ASD traits and Ras/MAPK signaling within this region of chromosome 7 that are proximal but do not represent overlap across analyses.

In addition, two other noteworthy loci (one between *KIRREL3* and *ETS1*, the other between *GATA3* and *CELF2*) overlap between top results from ASD epistasis and RASopathy modifier mapping and contain genes of particular interest. *KIRREL3* is a strong ASD candidate gene, as described above, and *ETS1* encodes an effector of Ras/MAPK signaling mediating cell migration and transcriptional activation expressed in astrocytes[103,104]. *GATA3* encodes a known Ras/MAPK interactor, as described above, and *CELF2* encodes CUGBP[105], Elav-like family member 2, a fetal and adult brain expressed regulator of alternative splicing also likely to be involved in mRNA editing and translation. Together, these loci overlapping with significant ASD epistasis results suggest that studying rare Mendelian disorders associated with symptoms of complex traits is a highly effective and relevant study design.

Further, some of the top results in the RASopathy modifier screen, although not meeting criteria for genome-wide significance, are within or adjacent to previously implicated ASD candidate genes. *MACROD2* has been identified near a GWAS association, shown to be associated with ASD traits in the general population, and described as part of the gene expression network of an independent ASD GWAS locus[106–108]. In addition, the *MACROD2* locus has been identified as associated with temporal lobe volume[109]. Likewise, *CDH10* was initially located near a GWAS association[110]. *RBFOX1* (or *A2BP1*) has been identified as disrupted by rare translocation or CNV in ASDs[111–113]. The encoded protein (FOX1) controls alternative splicing and transcription[114], including many other ASD candidate genes, and is thought to be a key regulator of neurodevelopment[115]. *DISC1*, known for identification via rare structural variant in schizophrenia, has also been associated with ASDs[78,112,116,117]. Additional genes listed in SFARIgene[89] adjacent to our top RASopathy social responsiveness QTL results include *CNTN5*, *ESRRB*, *GABRB1*, *GNB1L*, and *ICA1*.

Some clear Ras/MAPK related genes contain or are adjacent to top RASopathy modifier SNPs, as well. Primary RASopathy gene *SPRED1* is a negative regulator of the pathway; *MAP3K2* encodes MEK kinase 2; *RAPGEF2* encodes a Ras activator thought to control developmental neuronal migration in the cortex and formation of the corpus callosum[118,119]. *ELK3* encodes a transcription factor downstream of Ras/MAPK signaling; *ETS1* and *GATA3* are additional transcription factors with demonstrated relationships to Ras/MAPK signaling described above[120,121]. It is striking that the modifier-mapping approach in subjects ascertained for Mendelian disease appears to contain many plausible loci despite the small sample size compared with modern GWAS designs for case-control studies. However, it should be noted that proximity between a SNP and gene does not indicate a functional relationship, so further information would be needed to directly relate our identified loci from any analyses in this study to specific genes listed in tables or discussed here.

In summary, we have used a variety of approaches under a reverse pathway tactic of defining a relevant biological pathway and leveraging it to study a proposed genetic mechanism. In this case, we chose a pathway of interest based on human Mendelian disorders with overlapping symptoms shared by a genetically complex trait. Together, our strategies were successful in validating a role for the Ras/MAPK pathway in idiopathic ASDs, demonstrating highly significant enrichment for epistasis in ASDs, and identifying specific candidate loci interacting with the Ras/MAPK pathway pertinent to symptoms of ASDs. Our experimental data confirmed expression dysregulation of a gene within a convergently identified locus in RASopathy-specific neural cell lines. Future studies would be useful to follow up additional candidates identified by these approaches or extensions of them.

## Materials and Methods

### ASD datasets

ASD genotype data sets were collected from multiple sources. We obtained previously published genotype data as study investigators (UCSF-Weiss) or by application to AGRE, SSC, and dbGAP (AGP). Genotyping of each dataset was previously performed on Illumina or Affymetrix genotyping arrays as described in S6 Table. Diagnostic criteria were previously described in the respective references (S6 Table), but in summary, the Autism Diagnostic Interview-Revised (ADI-R)[122], and/or Autism Diagnostic Observation Schedule (ADOS)[123] criteria was used for diagnosis for the AGRE, AGP and SSC datasets, and clinician diagnosis for the UCSF-Weiss dataset. The ASD affected child and both parents were included in each study. All samples were anonymized for analysis.

### ASD dataset SNP quality control

Data preparation, quality control, and imputation were conducted as described previously in Mitra, I. *et al*. [124]. First, SNPs were filtered using PLINK[63,64] (see web resources) for Hardy-Weinberg equilibrium (HWE), call rate, minor allele frequency (MAF) and Mendel errors separately in each ASD dataset (S6 Table). Next, imputation was performed separately for each dataset using IMPUTE2[125] (see web resources), following the recommended pipeline. Lastly, each ASD dataset was combined together, and the following quality control steps were performed: SNPs with severe departure (*P* < 1.0x10^-6^) from HWE in Caucasian founders were removed; SNPs were removed if they had different MAF (*P* < 1.0x10^-6^) in Caucasians between multiple datasets; SNPs were removed if they had MAF < 1% in Caucasians, or MAF < 2% in the combined dataset. We excluded chromosome X. The final dataset for the analysis included 4,471,807 autosomal SNPs.

### Individual quality control for ASD datasets

Initial data preparation and quality control for ASD individuals was conducted for each dataset, as described previously in Mitra, I. *et al*. [124]. For each dataset, the following individual quality control filters were applied using PLINK[63,64]: genotyping rate, heterozygosity rate, verifying individual sex, verifying known relationships, removing individuals contributing to confounding relationship, and keeping one instance of individuals present in multiple studies. After combining the multiple datasets, sex and family structure were re-checked.

To avoid population stratification, we selected only Caucasian individuals for the analysis. Ancestry was determined using the first two principal components resulting from multidimensional scaling with PLINK[63,64] (—mds-plot option) (S8 Fig). A proband and both parents were required to fall within the Caucasian cluster for inclusion in the analysis. Only unrelated complete trios were used. The final dataset included 4,109 ASD affected cases (3,517 males and 592 females) with both parents. A QQ plot for main effect association is shown in S9 Fig. As our data were family-based, we used non-transmitted parental alleles, commonly known as pseudo-controls, generated by the—tucc option in PLINK[63,64] instead of unrelated healthy individuals. These 4,109 pseudo-controls are perfectly matched to cases for ancestry, thereby serving as a control for any population confounding.

### Creating the Ras/MAPK SNP set

We included the following genes in the Ras/MAPK pathway as the set of interest: *NF1*, *BRAF*, *SOS1*, *RASA2*, *RASA1*, *SOS2*, *MAPK1*, *MAP2K1*, *SPRED1*, *CBL*, *SHOC2*, *PTPN11*, *RAF1*, *KRAS*, *LZTR1*, *RIT1*, and *NRAS*. Genes with no SNPs represented within the ASD dataset (*HRAS*, *MAP2K2*, and *MAPK3*) could not be included. We extracted all SNPs within 5kb of each gene. 2,520 SNPs were included in the Ras/MAPK SNP set to be used for enrichment assessment in association and for the epistasis analysis (S1 Table).

### Ras/MAPK polymorphism enrichment in ASD association

Permutation testing procedures were implemented to establish significance of association signal enrichment in the Ras/MAPK SNP set. First, we performed a TDT (—tdt in PLINK[63,64]) in the 4,109 ASD trios to test for association. Then, at a given Benjamini and Hochberg’s FDR[126] (q = 0.2), we compared the percent of SNPs in the Ras/MAPK set (S1 Table) meeting this criterion in the TDT results to the empirical null distribution produced by permuted data. The FDR threshold was chosen to maximize power by minimizing instances of 0 or 100% of SNPs meeting the criterion, but is an arbitrary threshold not intended to indicate significance, only as a means of comparison with permuted data. At lower FDR thresholds, no SNPs are available to test, and beginning at FDR 0.2 approximately 10% of SNPs pass the threshold, which is sufficient for testing enrichment (S7 Table). To generate each of 100 permuted gene sets, a random gene was selected from 100 RefSeq (see web resources) genes with the most similar size (using the longest transcript) to each Ras/MAPK gene (S1 Table) and compiled into a SNP set using the same procedure (all SNPs within 5kb). These sets appear well matched to the Ras/MAPK set, as they have similar numbers of SNPs (Ras/MAPK 2,520; permutation median 2,528) and allele frequency (Ras/MAPK average 0.18; permutation average 0.19). The permuted gene lists were analyzed with the same protocol as the Ras/MAPK set to produce the null distribution for comparison. The empirical *P*-value was calculated as the proportion of results from the null distribution equal to or greater than the results from the Ras/MAPK set[127].

### Epistasis Ras/MAPK ‘set-by-all’ case-only test

For the epistasis analysis, the dataset was comprised of 4,109 ASD affected cases and 4,471,807 SNPs. We used the—fast-epistasis test with the case-only and set-by-all options implemented in PLINK[63,64] to perform pairwise epistasis tests between each SNP in the defined Ras/MAPK SNP set (N = 2,520 SNPs) and SNPs across the autosomal genome. This epistasis test is performed by testing an allelic odds ratio, based on collapsing the 4N independent alleles observed at two loci in a sample of N individuals into a 2x2 table, so the allele (not the individual or haplotype) is the unit of analysis. The four cells are (a) 4*AABB+2*AABb+2*AaBB+AaBb, (b) 4*AAbb+2*AABb+2*Aabb+AaBb, (c) 4*aaBB+2*aaBb+2*AaBB+AaBb, (d) 4*aabb+2*aaBb+2*Aabb+AaBb. The odds ratio is then estimated as ad/bc with variance 1/a+1/b+1/c+1/d. This test follows a standard normal distribution under the multiplicative model of no interaction. Appropriate type I error rates have been observed in simulation and power is equivalent to a logistic regression test for epistasis. The correlation with a logistic regression analysis is high (r = 0.995) [63],[64].

Two SNPs on the same chromosome were excluded from consideration in order to conservatively eliminate linkage disequilibrium or effects of rare variants. We calculated a genome-wide significance threshold (*P* = 7.6x10^-10^) accounting for multiple LD groups per Ras/MAPK gene and the proportion of the genome tested for each genome-wide epistasis screen (calculations shown in S2 Table). We also utilized a gene-based significance threshold set by dividing the GWAS significance threshold (*P* = 5.0x10^-8^) by the number of genes in the Ras/MAPK set for an approximate independent hypothesis-testing estimate (*P* = 2.9x10^-9^), as genes were the functional unit of the set-based testing. Because we use a case-only approach with similar properties to identifying significant correlation, we have calculated power based on correlation statistics. In this instance, for our stringent *P*-value threshold of 7.6 x 10^−10^, we have >80% power to detect r^2^ = 0.11 with our sample size 4,109.

In order to rule out nonspecific effects and control for false-positives, we performed the same epistasis analysis on the 4,109 matched pseudo-controls as a negative control. To test if the most significant epistasis results between the ASD cases and ASD pseudo-controls were significantly different, we performed a 2x2 chi-square test using the number of SNPs meeting a given significance threshold in cases compared with pseudo-controls. We conducted a second negative control analysis to rule out results due to interactions associated with viability by using 4,109 parents that were of the same sex as the ASD cases. In addition, we performed epistasis testing in our sample for a permuted SNP set included in a non-candidate pathway selected set as a negative control and for Ras/MAPK set in a homogeneous set of cases (N = 1,136) and matched unaffected siblings (N = 1,136) from unique families from the SSC dataset as a positive control. For both, we measured odds ratios at varying *P*-value thresholds down to *P* < 10^−4^ (S2 and S3 Figs).

### Trio correlation test

To statistically validate the results from the case-only epistasis test, we used an independent test, called the trio correlation test[128].The trio correlation test leverages information from the parental genotypes to compute the expected distribution of the offspring genotypes then used in a correlation test. This test was provided to us as an R script (see web resources). To individually test candidate SNP pairs for interaction, we tested the nominally significant interaction results (*P* < 1.0x10^-6^) from the PLINK[63,64] epistasis test. To confirm the absence of false-positive results, we performed a Spearman correlation test in R (see web resources) between the *P*-values of the nominally significant PLINK[63,64] epistasis test results (*P* < 1.0x10^-6^) and their corresponding *P*-value from the trio correlation test (S4 Fig).

### RASopathy dataset

We have previously described in detail the recruitment and phenotype data collection in RASopathy subjects[56]. In summary, patients with a physician (medical geneticist or neurologist) confirmed NF1, NS, CS, CFC diagnosis were included in the study. We recruited subjects from the NF/RAS Pathway Genetics Clinic UCSF, UCSF NF Symposium, RASopathy support groups (NF, Inc., Children’s Tumor Foundation, Noonan Syndrome Foundation, CFC International, Costello Syndrome Family Support Network, and Costello Kids), and three national RASopathy family meetings (Chicago, Illinois, USA, July 2011; Berkeley, California, USA, July 2009; Orlando, Florida, USA, August 2013). In addition, NF1 patients were recruited at University of California, Los Angeles (UCLA) through online postings (NF, Inc., and Children's Tumor Foundation), and the Neurofibromatosis and Neurocutaneous Disorders clinic at the Children's Hospital, Los Angeles. We enrolled the unaffected siblings of RASopathy subjects as controls.

To measure ASD symptoms, we used the SRS questionnaire[129]. The SRS questionnaire is a quantitative and continuous measure of social ability. Parents or persons well-acquainted with the study participant answered the 65-item SRS questionnaire regarding traits characteristic of ASD. Following the SRS manual, we calculated the raw score for each individual, and then calculated the sex-normalized T-scores. A total of 257 RASopathy patients and 142 RASopathy-unaffected full siblings of RASopathy patients with SRS phenotype data were recruited. Further detailed information about the SRS questionnaire data for the sample population can be found in Adviento *et al*. (2014)[56].

### RASopathy dataset genotyping and quality control

All participants provided blood or saliva samples for DNA extraction. Blood samples were collected by venipuncture using standard procedures. Saliva samples of families involved in the study were collected by mail or in person at family meetings. Participants provided saliva samples using the Oragene Discover kit (OGR-250 for children and OGR-500 for adults) by DNA Genotek (see web resources). DNA was extracted using the manufacturer’s standard protocol. All specimens were anonymized for analysis.

All DNA samples were genotyped in the Genomics Core Facility (GCF) of UCSF on the Affymetrix Axiom EUR array following standard manufacturer protocols. The Axiom EUR array contains approximately 675,000 SNPs across the genome[130]. Genotype calling was performed using Axiom GT1 algorithm as part of the Affymetrix Genotyping Console^™^ (GTC) Software (see web resources). For analysis, we used samples that had a dish QC (probe intensity) threshold greater than 82% and a genotype call rate greater than 97%. Additional quality control procedures were performed in PLINK[63,64]. Identification of samples was validated based on sex and familial relationships, using pairwise identity by decent (IBD) estimation (—genome). Samples failing quality control checks, including incorrect sex (—check-sex), excessive heterozygosity (—het), and other indicators of DNA contamination were removed. One sample was selected for analysis from monozygotic twin pairs or duplicate samples. To ensure that within each group all subjects were unrelated, a maximum of one person per family was selected to be in each of the control sibling, CFC, CS, NF1, and NS groups. SNPs were removed based on the following quality filters: ≥ 5% missing rate, and Hardy-Weinberg equilibrium *P* ≤10^−4^. The final dataset used for analysis included 658,746 SNPs with a 99.70% genotyping rate. The final individuals included were 209 RASopathy subjects (49 CFC, 50 CS, 60 NF1, 50 NS) and 84 control siblings.

### Data analysis in RASopathy dataset

We performed a multidimensional scaling analysis of genome-wide pairwise identity-by-state (IBS) distances in PLINK[63,64] for all individuals in the dataset. We used the first five dimensions as covariates in the analysis to correct for population stratification and batch effects. To accurately compare between the RASopathy and control sibling groups, we scaled the T-scores within each group (CFC, CS, NF1, NS, and sibling) so that the mean of the values was 0 and variance was 1, and excluded outlier values greater or less than 3 standard deviations (SD) from the mean (S5 Fig). For each group (CFC, CS, NF1, NS, sibling), we performed QTL mapping by implementing in PLINK[63,64] a linear regression analysis using the scaled SRS scores as a quantitative trait (—linear). This resulted in the multi-linear regression model Y = b0 + b1*ADD + b2*COV1 + b3*COV2… bn*COV5 + e. To analyze the RASopathy groups together (CFC, CS, NF1, and NS) with greater statistical power, we used METASOFT[131] (see web resources) to conduct a random effects meta-analysis using Han and Eskin's random effects model[131]. We also report the Cochran’s Q statistic, calculated using METASOFT[131], to analyze heterogeneity between RASopathy groups. The data underlying the top six potential modifiers are graphically represented in S6 Fig, by boxplot (MAF>0.05) or distribution (MAF≤ 0.05).

### RNA extraction from induced pluripotent stem cell-derived neural cells

To induce neural differentiation, free floating induced pluripotent stem cell (iPSC) aggregates were formed for 24 hours in mTeSR1 (Stemcell Technologies) and then switched to a Neural medium [DMEM/F12 (Invitrogen), N2 supplement (Invitrogen), MEM-NEAA (Gibco) and 2 μg/ml Heparin (Sigma-Aldrich)] with media exchange every other day[132]. To promote neural induction, the small molecules SB431542 (5 μM, Stemgent) and LDN-193189 (0.25 μM, Stemgent) were added for 48 hours. On day 3, aggregates were attached to 6 well plates and cultured in neural media for an additional week during which rosettes appeared in the colonies. On day 11, neuroepithelial cells in the center of the colonies were mechanically removed and kept as free floating aggregates. At day 25 of neural differentiation, neurospheres were dissociated into single cells using Accutase (Stem Cell Technologies) and cultured as monolayer neural progenitor cells (NPCs). NPCs were plated into poly-ornithine/laminin -coated plates at 50,000 cells/cm2 and fed with forebrain neuronal medium [Neurobasal medium (Invitrogen), supplemented with N2 supplement (Invitrogen), and B27 supplement (Invitrogen)[132]. Cells were fed twice a week and RNA samples were extracted at 5 weeks after plating. Total RNA was isolated using RNeasy Mini kit (Qiagen) according to manufacturer’s instruction.

### Reverse transcription reactions and quantitative real time PCR

Complementary DNA (cDNA) was produced from 1 μg of total RNA using High Capacity RNA-to-cDNA Kit (Life technologies). The qRT-PCR assay was performed using approximately 20 ng of cDNA and Taqman gene expression master mix in a QuantStudio^™^ 6 Flex Real-Time PCR System (Applied Biosystems). Expression level was determined by relative quantification in comparison to the endogenous control gene *GUSB*. Expression of each target gene (*ELMO1*, *GPR141*, *NME8*, *SFRP4*, *EPDR1*, and *STARD3NL)* was assessed relative to a control sample (comparative Ct method). Samples were run in technical triplicates, and the threshold suggested by the instrument software was used (after visual confirmation) to calculate the Ct. Outlier replicate samples were excluded from analysis. The Taqman probes used in this study are summarized in S8 Table.

## Ethics Statement

All subjects or their legal guardians gave written informed consent. This study was approved by the institutional review boards of UCSF Human Research Protection Program (CHR #10–02794) and University of California, Los Angeles (UCLA, IRB#10–000518).

## Web Resources

Affymetrix Genotyping Console^™^ (GTC) Software: http://www.affymetrix.com/estore/browse/level_seven_software_products_only.jsp?productId=131535#1_1

Haploview: https://www.broadinstitute.org/scientific-community/science/programs/medical-and-population-genetics/haploview/haploview

IMPUTE2: https://mathgen.stats.ox.ac.uk/impute/impute_v2.html

LocusZoom: http://locuszoom.sph.umich.edu/locuszoom/

METASOFT: http://genetics.cs.ucla.edu/meta

Oragene Discover kit OGR-250 by DNA Genotek: http://www.dnagenotek.com/US/products/OGR250.html

Oragene Discover kit OGR-500 by DNA Genotek: http://www.dnagenotek.com/US/products/OGR500.html

PLINK: http://pngu.mgh.harvard.edu/~purcell/plink/index.shtml

R—A language and environment for statistical computing: http://www.R-project.org/

RefSeq Genes Database–UCSC: http://hgdownload.cse.ucsc.edu/goldenPath/hg19/database/knownToRefSeq.txt.gz

SFARIgene: https://gene.sfari.org/autdb/Welcome.do

Trio Correlation Test R script: https://github.com/BrunildaBalliu/TrioEpi

## Accession Numbers

The accession number for the UCSF RASopathies social responsiveness and genotype data reported in this paper is The National Database for Autism Research (NDAR) ID 1966.

## Supporting Information

S1 Table Ras/MAPK SNP set. (PDF)

S2 Table Calculation of multiple testing thresholds. (PDF)

S3 Table Epistasis data for top SNPs. (PDF)

S4 Table Main effects for epistatic SNPs. (PDF)

S5 Table Results of qRT-PCR. (PDF)

S6 Table ASD datasets. (PDF)

S7 Table Association signal at varying FDR thresholds. (PDF)

S8 Table Table of Taqman probes. (PDF)

S1 Fig Most significant epistasis results in ASD. (PDF)

S2 Fig Epistasis for a non-candidate pathway selected set. (PDF)

S3 Fig Epistasis in sibling-paired SSC dataset. (PDF)

S4 Fig Correlation plot of top Ras/MAPK—ASD epistasis results. (PDF)

S5 Fig Distribution of social responsiveness scores per RASopathy. (PDF)

S6 Fig Distribution of social responsiveness scores for top six candidate modifiers. (PDF)

S7 Fig Neural cell lines. (PDF)

S8 Fig Population structure of ASD dataset. (PDF)

S9 Fig QQ-plot of ASD association. (PDF)
