# Supplementary material for: Reverse Pathway Genetic Approach Identifies Epistasis in Autism Spectrum Disorders
Source: PLoS Genet. 2017 Jan 11;13(1):e1006516. doi: 10.1371/journal.pgen.1006516 (PMC5226683; doi:10.1371/journal.pgen.1006516)
Supplement: S1 Table — (PDF) [file pgen.1006516.s001.pdf]

Table S1: Ras/MAPK SNP set. The genes associated with the Ras/MAPK pathway that were used in the analysis are listed in the table. For each gene, the table lists the following in order of columns: HUGO gene name, associated RASopathy disorder or pathway role, chromosome, position (reference version hg19), region length with +/-5 kb flanking the gene, and total number of SNPs overlapping with the ASD dataset. CFC: cardiofaciocutaneous syndrome; NS: Noonan syndrome; CS: Costello syndrome; NF1: neurofibromatosis type 1; CM-AVM: Capillary malformation-arteriovenous malformation syndrome; effector – protein carrying out cellular signal transduction.

gene	RASopathy	chromosome	position	length (bp)	total SNPs
BRAF	CFC, NS	7	140,433,813-140,624,564	200,751	267
CBL	NS	11	119,076,986-119,178,859	111,873	74
HRAS	CS	11	532,242-535,550	13,308	0
KRAS	CFC, NS	12	25,358,180-25,403,854	55,674	139
LZTR1	NS	22	21,336,558-21,353,326	26,768	1
MAP2K1	CFC, NS	15	66,679,211-66,783,882	114,671	276
MAP2K2	CFC	19	4,090,320-4,124,126	43,806	0
MAPK1	[effector]	22	22,113,947-22,221,970	118,023	218
MAPK3	[effector]	16	30,125,426-30,134,630	19,204	0
NF1	NF1	17	29,421,945-29,704,695	292,750	249
NRAS	NS	1	115,247,085-115,259,515	22,430	22
PTPN11	NS	12	112,856,536-112,947,717	101,181	21
RAF1	NS	3	12,625,100-12,705,700	90,600	187
RASA1	CM-AVM	5	86,564,070-86,687,743	133,673	65
RASA2	NS	3	141,205,926-141,331,197	135,271	86
RIT1	NS	1	155,867,599-155,880,706	23,107	28

SHOC2	NS	10	112,679,301-112,773,425	104,124	164
SOS1	NS	2	39,208,690-39,347,604	148,914	176
SOS2	NS	14	50,583,846-50,698,099	124,253	240
SPRED1	NF1-like syndrome	15	38545,052-38,649,450	114,398	307
