# Supplementary material for: Reverse Pathway Genetic Approach Identifies Epistasis in Autism Spectrum Disorders
Source: PLoS Genet. 2017 Jan 11;13(1):e1006516. doi: 10.1371/journal.pgen.1006516 (PMC5226683; doi:10.1371/journal.pgen.1006516)
Supplement: S2 Table — (PDF) [file pgen.1006516.s002.pdf]

Table S2. Calculation of multiple testing thresholds. The Ras/MAPK SNP set is shown, including gene, chromosome positions, total SNPs, LD-independent SNPs, and proportion of the genome screened for epistasis (subtracting the X chromosome and gene chromosome proportion from approximate 3 billion bp genome). The last row indicates the threshold calculated by multiplying 5 x 10^{-8} by the gene or screening proportion x LD-independent SNPs proportion total for gene-based and genome-wide independent thresholds, respectively.

gene	CHR	START	END	N total SNPs	N SNPs (R^2<0.2)	screening proportion (%)
BRAF	7	140428813	140629564	267	4	89.5
CBL	11	119071986	119183859	74	2	90.3
KRAS	12	25353180	25408854	139	8	90.4
LZTR1	22	21331558	21358326	1	1	93.1
MAP2K1	15	66674211	66788882	276	8	91.4
MAPK1	22	22108947	22226970	218	5	93.1
NF1	17	29416945	29709695	249	4	92
NRAS	1	115242085	115264515	22	4	86.5
PTPN11	12	112851536	112952717	21	4	90.4
RAF1	3	12620100	12710700	187	4	88.2
RASA1	5	86559070	86692743	65	6	88.8
RASA2	3	141200926	141336197	86	4	88.2
RIT1	1	155862599	155885706	28	2	86.5
SHOC2	10	112674301	112778425	164	5	90.3
SOS1	2	39203690	39352604	176	3	86.7
SOS2	14	50578846	50703099	240	4	91.2
SPRED1	15	38540052	38654450	307	5	91.4
**17 genes**				**2520 SNPs**	**73 ind.**	**65.72 ind x prop**
2.9 x 10^{-9}						7.6 x 10^{-10}

Abbreviations: CHR chromosome; START start position (bp); END end position (bp)
