# Supplementary material for: Reverse Pathway Genetic Approach Identifies Epistasis in Autism Spectrum Disorders
Source: PLoS Genet. 2017 Jan 11;13(1):e1006516. doi: 10.1371/journal.pgen.1006516 (PMC5226683; doi:10.1371/journal.pgen.1006516)
Supplement: S3 Table — (PDF) [file pgen.1006516.s003.pdf]

**Table S3. Epistasis data for top SNPs.** For each of the top epistasis results in Table 1, we show the epistasis data here. The SNP rsIDs are shown with the Ras/MAPK (RMK) SNP first and epistatic (EPI) SNP second. Genotype abbreviations are: HOM – homozygous for major allele; hom – homozygous for minor allele; Het – heterozygous. The observed counts for each genotype-combination are shown in the same order as SNPs (e.g. HOM-Het represents RMK SNP homozygous for the major allele and EPI SNP heterozygous), with expected counts and pseudo-control counts (respectively) displayed in brackets. Cells with count of more than 5 observed or expected are **bolded** if at least 2X excess is observed in cases compared with expectation and are shown in *italics* if at least 0.5X decrease is observed in cases compared with expectation. Asterisks are used to show pseudo-control counts deviating 2-fold from expectation in either direction.

| RMK-EPI SNPs | HOM-HOM | HOM-Het | Het-HOM | Het-Het | HOM-hom | hom-HOM | Het-hom | hom-Het | hom-hom |
|---|---|---|---|---|---|---|---|---|---|
| rs713365-rs57173428 | 3830<br>[3815/3820] | 93<br>[105/118] | 126<br>[141/124] | **14**<br>**[4/3]** | 1<br>[1/1] | 2<br>[1/1] | 1<br>[0/0] | 0<br>[0/0] | 0<br>[0/0] |
| rs7133635-rs17110869 | 3817<br>[3805/3807] | 92<br>[104/120] | 128<br>[140/123] | **14**<br>**[4/3]** | 1<br>[1/1] | 2<br>[1/1] | 1<br>[0/0] | 0<br>[0/0] | 0<br>[0/0] |
| rs11913063-rs28459694 | 3377<br>[3361/3352] | 78<br>[97/107] | 307<br>[315/314] | **23**<br>**[9/9]** | 4<br>[1/1] | *0*<br>*[7/7]* | 1<br>[0/0] | 1<br>[0/1] | 0<br>[0/0] |
| rs62487902-rs72690923 | 3042<br>[3004/2965] | 185<br>[212/216] | 453<br>[490/536] | **61**<br>**[34/28]** | **19**<br>**[4/3]** | *1*<br>*[20/15]* | 1<br>[1/2] | 4<br>[1/1] | 0<br>[0/0] |
| rs1733826-rs12239450 | 2792<br>[2756/2762] | 354<br>[387/359] | 263<br>[295/313] | **72**<br>**[41/47]** | 11<br>[14/14] | 7<br>[8/9] | 3<br>[1/0] | 2<br>[1/0] | 0<br>[0/0] |
| rs73398437-rs62168052 | 3292<br>[3269/3251] | 154<br>[179/178] | 170<br>[189/206] | **30**<br>**[10/13]** | 5<br>[2/1] | 2<br>[3/4] | 0<br>[0/0] | 0<br>[0/0] | 0<br>[0/0] |
| chr7:140625707:I-rs1318299 | 3248<br>[3237/3230] | 65<br>[79/78] | 381<br>[392/405] | **26**<br>**[10/7]** | 0<br>[0/0] | 10<br>[12/10] | 0<br>[0/0] | 1<br>[0/1] | 0<br>[0/0] |
| rs74644204-rs149565205 | 3448<br>[3438/3462] | 83<br>[93/66] | 160<br>[173/168] | **17**<br>**[5/6]** | 0<br>[1/2] | 2<br>[2/7*] | 0<br>[0/0] | 1<br>[0/0] | 0<br>[0/0] |
| rs7714386-rs56667163 | 3509<br>[3490/3507] | 413<br>[423/417] | 103<br>[130/124] | **37**<br>**[16/11]** | *6*<br>*[13/13]* | 3<br>[1/0] | 2<br>[0/1] | 0<br>[0/0] | 0<br>[0/0] |
| rs17879775-rs11925140 | 3524<br>[3607/3514] | 115<br>[129/142] | **183**<br>**[95/75]** | **14**<br>**[3/3]** | 0<br>[1/2] | 0<br>[1/0] | 0<br>[0/0] | 0<br>[0/0] | 0<br>[0/0] |
| chr2:39208512:I-rs304654 | 3326<br>[3302/3317] | 73<br>[92/90] | 448<br>[474/465] | **31**<br>**[13/10]** | 0<br>[1/18*] | 19<br>[17/1*] | 1<br>[0/0] | 2<br>[0/0] | 0<br>[0/0] |
| chr15:38591893:D-rs73760016 | 3432<br>[3407/3422] | 200<br>[225/222] | 189<br>[206/191] | **33**<br>**[14/15]** | 2<br>[4/3] | 2<br>[3/4] | 1<br>[0/1] | 0<br>[0/1] | 0<br>[0/0] |
| rs117147554-rs80214471 | 3289<br>[3277/3263] | 129<br>[143/164] | 152<br>[163/158] | **20**<br>**[7/6]** | 3<br>[2/0] | 0<br>[2/4] | 1<br>[0/0] | 1<br>[0/0] | 0<br>[0/0] |
| rs75961317-rs143823697 | 3291<br>[3271/3293] | 185<br>[203/207] | 219<br>[241/220] | **34**<br>**[15/14]** | 4<br>[3/2] | 3<br>[4/2] | 0<br>[0/0] | 2<br>[0/0] | 0<br>[0/0] |
| rs75087565-rs254700 | 3412<br>[3399/3399] | 73<br>[84/75] | 172<br>[189/200] | **17**<br>**[5/3]** | 1<br>[1/0] | 5<br>[3/3] | 0<br>[0/0] | 0<br>[0/0] | 0<br>[0/0] |
| rs58347743-rs80179511 | 2426<br>[2382/2414] | 310<br>[345/352] | 660<br>[694/658] | 135<br>[101/103] | *5*<br>*[12/9]* | 36<br>[51/46] | 7<br>[4/5] | **15**<br>**[7/9]** | 2<br>[0/0] |
| rs41280637-rs114617777 | 3248<br>[3227/3247] | 369<br>[389/384] | 148<br>[171/150] | **43**<br>**[21/23]** | 8<br>[12/15] | 3<br>[2/3] | 2<br>[1/0] | 1<br>[0/0] | 0<br>[0/0] |
| rs11818771-chr6:80625529:D | 2679<br>[2638/2615] | 668<br>[699/716] | 214<br>[257/263] | 95<br>[68/69] | 42<br>[46/48] | 6<br>[6/5] | **12**<br>**[5/4]** | 5<br>[2/1] | 0<br>[0/0] |
| rs9672789-rs113552799 | 2709<br>[2678/2667] | 219<br>[255/239] | 453<br>[477/498] | 69<br>[45/44] | 5<br>[6/5] | 19<br>[21/29] | 4<br>[1/1] | **7**<br>**[2/2]** | 0<br>[0/0] |
| rs12309312-rs118078508 | 3508<br>[3498/3477] | 164<br>[176/176] | 77<br>[86/109] | **16**<br>**[4/2]** | 2<br>[2/2] | 0<br>[1/0] | 0<br>[0/1] | 0<br>[0/0] | 0<br>[0/0] |
| rs3093853-rs114490548 | 3530<br>[3505/3466] | 142<br>[168/177] | 169<br>[190/218] | **26**<br>**[9/11]** | 4<br>[2/1] | 4<br>[3/3] | 0<br>[0/0] | 1<br>[0/0] | 0<br>[0/0] |
| rs114670618-rs2043732 | 3184<br>[3162/3179] | 279<br>[305/273] | 165<br>[184/195] | **38**<br>**[18/20]** | 10<br>[7/5] | 1<br>[3/6*] | 2<br>[0/0] | 1<br>[0/1] | 0<br>[0/0] |
| rs117672593-rs73688732 | 3347<br>[3333/3340] | 128<br>[142/133] | 213<br>[229/235] | **26**<br>**[10/9]** | 0<br>[2/1] | 4<br>[4/1] | 1<br>[0/0] | 0<br>[0/0] | 0<br>[0/0] |
| rs6948377-rs1826547 | 3158<br>[3137/3138] | **334**<br>**[120/136]** | *99*<br>*[352/335]* | **29**<br>**[13/13]** | 1<br>[1/2] | 9<br>[10/9] | 2<br>[0/0] | 2<br>[0/1] | 0<br>[0/0] |
| rs116702532-rs41274082 | 3548<br>[3534/3521] | 180<br>[194/186] | 119<br>[135/155] | **24**<br>**[7/6]** | 2<br>[3/4] | 2<br>[1/3] | 0<br>[0/0] | 0<br>[0/0] | 0<br>[0/0] |
| rs61761074-rs140695911 | 3256<br>[3243/3245] | 170<br>[183/82] | 156<br>[170/170] | **25**<br>**[10/10]** | 1<br>[3/3] | 1<br>[2/1] | 2<br>[0/0] | 0<br>[0/0] | 0<br>[0/0] |

| | | | | | | | | | |
|---|---|---|---|---|---|---|---|---|---|
| rs75217189-rs192196641 | 3372 [3358/3332] | 262 [281/317] | 150 [163/144] | **33 [14/18]** | 4 [6/10] | 1 [2/2] | 1 [0/1] | 1 [0/0] | 0 [0/0] |
| rs75756950-rs2765709 | 2824 [2779/2786] | 451 [483/456] | 285 [335/340] | 91 [58/66] | 15 [21/24] | 14 [10/15] | 5 [3/2] | **5 [2/1]** | 0 [0/0] |
| rs75217189-rs11255742 | 3358 [3345/3319] | 231 [246/281] | 149 [163/145] | **29 [12/17]** | 2 [5/8] | 1 [2/2] | 1 [0/0] | 1 [0/0] | 0 [0/0] |
| rs72417307-rs2441690 | 3339 [3302/3273] | 380 [423/404] | 223 [252/285] | 58 [32/44] | 18 [14/17] | 4 [5/5] | **6 [1/1]** | 1 [1/0] | 0 [0/0] |
| rs2123876-chr12:21624694:D | 2961 [2914/2922] | 529 [575/569] | 374 [419/419] | 123 [83/80] | 28 [28/29] | 13 [15/18] | 6 [4/2] | **7 [3/1]** | 0 [0/1] |
| rs117834974-rs12582581 | 3612 [3600/3610] | 163 [132/124] | 119 [177/178] | **21 [6/4]** | 2 [1/1] | 2 [2/1] | 0 [0/1] | 0 [0/0] | 0 [0/0] |
| rs17879775-rs78762238 | 3385 [3374/3404] | 130 [142/128] | 80 [89/73] | **14 [4/5]** | 1 [1/1] | 0 [1/0] | 1 [0/0] | 0 [0/0] | 0 [0/0] |
| rs1357384-chr13:78700408:D | 3594 [3582/3579] | 94 [106/112] | 144 [156/157] | **17 [5/3]** | 1 [1/0] | 2 [2/1] | 0 [0/0] | 0 [0/0] | 0 [0/0] |
| rs75087565-rs73475884 | 3050 [3017/3067] | 417 [447/393] | 138 [168/175] | **49 [25/27]** | 16 [17/12] | 4 [2/3] | 2 [1/0] | 1 [0/0] | 0 [0/0] |
| rs75087565-rs55942942 | 3047 [3015/3065] | 419 [449/393] | 139 [167/176] | **49 [25/27]** | 16 [17/12] | 4 [2/3] | 2 [1/0] | 1 [0/0] | 0 [0/0] |
| rs13083303-chr15:71697182:D | 2895 [2866/2862] | 95 [126/104] | 842 [870/875] | **71 [38/37]** | 0 [1/1] | 63 [66/88] | 0 [0/1] | 5 [3/3] | 0 [0/0] |
| rs7714386-rs60709797 | 3573 [3555/3538] | 113 [174/200] | 160 [132/118] | **20 [6/10]** | 2 [2/4] | 2 [1/0] | 0 [0/1] | 1 [0/0] | 0 [0/0] |
| rs7714386-rs58413939 | 3572 [3554/3543] | 175 [188/209] | 113 [132/119] | **20 [7/9]** | 2 [2/4] | 2 [1/0] | 0 [0/1] | 1 [0/0] | 0 [0/0] |
| rs297122-rs4128728 | 3439 [3418/3399] | 126 [145/163] | 275 [293/293] | **25 [12/15]** | 3 [2/1] | *3 [6/4]* | 0 [0/0] | **5 [0/1]** | 0 [0/0] |
