# Supplementary material for: Reverse Pathway Genetic Approach Identifies Epistasis in Autism Spectrum Disorders
Source: PLoS Genet. 2017 Jan 11;13(1):e1006516. doi: 10.1371/journal.pgen.1006516 (PMC5226683; doi:10.1371/journal.pgen.1006516)
Supplement: S4 Table — (PDF) [file pgen.1006516.s004.pdf]

Table S4. Main effects for epistatic SNPs

SNP	CHR	position	gene	MAF	OR	P
chr15:38591893:D	15	38591893	SPRED1	0.03	1.05	0.59
chr2:39208512:I	2	39208512	SOS1	0.07	1.03	0.59
chr7:140625707:I	7	140625707	BRAF	0.06	0.98	0.73
rs114670618	3	141261220	RASA2	0.03	0.90	0.29
rs116702532	15	66676315	MAP2K1	0.02	0.87	0.21
rs117147554	15	66729976	MAP2K1	0.02	1.02	0.83
rs117672593	15	66720112	MAP2K1	0.03	1.00	0.96
rs117834974	11	119155649	CBL	0.02	1.03	0.79
rs11818771	10	112727826	SHOC2	0.05	0.98	0.82
rs11913063	22	22211605	MAPK1	0.05	1.00	1.00
rs12309312	12	112879955	PTPN11	0.01	0.82	0.14
rs13083303	3	12638696	RAF1	0.14	0.95	0.24
rs1357384	15	38596084	SPRED1	0.02	1.01	0.91
rs1733826	7	140495573	BRAF	0.05	0.91	0.21
rs17879775	17	29686758	NF1	0.01	1.23	0.17
rs2123876	2	39255873	SOS1	0.07	1.01	0.93
rs297122	2	39309141	SOS1	0.04	0.96	0.64
rs3093853	5	86690555	RASA1	0.03	0.86	0.12
rs41280637	2	39234432	SOS1	0.02	1.12	0.28
rs58347743	3	12622058	RAF1	0.12	1.05	0.33
rs61761074	12	25397593	KRAS	0.03	1.02	0.88
rs62487902	7	140438422	BRAF	0.08	0.93	0.22
rs6948377	7	140584262	BRAF	0.05	1.07	0.38
rs7133635	12	25388290	KRAS	0.02	1.15	0.25
rs72417307	22	22210145	MAPK1	0.04	0.87	0.08
rs73398437	15	38600123	SPRED1	0.03	0.89	0.24
rs74644204	12	112862991	PTPN11	0.03	0.97	0.79
rs75087565	10	112692665	SHOC2	0.03	0.95	0.61
rs75217189	15	66788118	MAP2K1	0.02	1.12	0.29
rs75756950	22	22126824	MAPK1	0.06	0.95	0.51
rs75961317	22	22215130	MAPK1	0.03	1.13	0.16
rs7714386	5	86634506	RASA1	0.02	1.11	0.40

rs9672789	15	66713633	MAP2K1	0.08	0.92	0.18
rs12239450	1	47933492	FOXD2 / TRABD2B	0.09	1.07	0.33
rs57173428	1	55100883	ACOT11 / TTC4	0.02	0.90	0.43
rs17110869	1	55108237	MROH7	0.02	0.88	0.32
rs72690923	1	106012196	AMY1B / PRMT6	0.03	1.01	0.93
rs28459694	1	158355190	CD1E / OR10T2	0.03	0.90	0.43
rs62168052	2	135088091	MGAT5	0.02	0.98	0.88
rs1318299	2	220283259	DES	0.02	1.08	0.61
rs149565205	3	6539899	EDEM1 / GRM7	0.03	1.27	0.10
rs11925140	3	112842425	GTPBP8 / BOC	0.02	0.89	0.32
rs56667163	3	136773620	IL20RB / SOX14	0.06	1.02	0.74
rs304654	4	124123184	SPATA5	0.02	1.07	0.62
rs73760016	5	19469048	BASP1 / CDH18	0.03	0.98	0.86
rs80214471	5	66139877	MAST4	0.03	0.89	0.29
rs254700	5	134103554	DDX46	0.02	1.19	0.24
rs143823697	5	134412896	PITX1 / H2AFY	0.03	1.00	0.96
chr6:80625529:D	6	80625529	ELOVL4	0.13	0.98	0.67
rs114617777	6	88085754	SMIM8 / CFAP206	0.06	1.00	0.94
rs113552799	6	123947624	TRDN	0.04	1.06	0.47
rs80179511	6	154274998	RGS17 / OPRM1	0.06	0.99	0.90
rs118078508	6	154999201	CNKSR3 / SCAF8	0.02	1.00	1.00
rs73688732	7	31068898	GHRHR / ADCYAP1R1	0.02	1.05	0.65
rs114490548	7	38139570	EPDR1 / STARD3NL	0.02	0.95	0.64
rs2043732	7	51192161	COBL	0.06	1.12	0.15
rs1826547	8	27414024	EPHX2 / SCARA3	0.03	0.90	0.35
**rs192196641**	**10**	**8587332**	**GATA3 / CELF2**	**0.04**	**0.85**	**0.04**
**rs11255742**	**10**	**8598746**	**KRT8P16**	**0.03**	**0.85**	**0.05**
rs2765709	10	25507753	GPR158	0.10	1.03	0.66
rs140695911	10	29495250	BAMBI / LYZL1	0.02	1.00	0.96
rs41274082	10	65381281	REEP3	0.02	1.05	0.62
rs2441690	11	127107790	KIRREL3 / ETS1	0.05	1.01	0.87
rs12582581	12	511258	CCDC77	0.02	1.09	0.47
chr12:21624694:D	12	21624694	RECQL	0.10	1.02	0.79
**rs73475884**	**13**	**35080882**	**RFC3 / NBEA**	**0.07**	**1.19**	**0.01**
**rs55942942**	**13**	**35095658**	**RFC3**	**0.07**	**1.19**	**0.01**

rs78762238	13	64302181	PCDH20/ PCDH9	0.03	1.10	0.41
chr13:78700408:D	13	78700408	EDNRB / POU4F1	0.02	0.97	0.84
rs58413939	15	23003403	CYFIP1	0.04	0.87	0.17
rs60709797	15	23004989	NIPA2	0.03	0.84	0.08
chr15:71697182:D	15	71697182	THSD4	0.02	1.16	0.20
rs4128728	18	49575401	MEX3C / DCC	0.03	0.89	0.30

Abbreviations: CHR chromosome; MAF minor allele frequency; OR odd ratio; P p-value; SNPs with P-value< 0.05 are in bold
