# Supplementary material for: Reverse Pathway Genetic Approach Identifies Epistasis in Autism Spectrum Disorders
Source: PLoS Genet. 2017 Jan 11;13(1):e1006516. doi: 10.1371/journal.pgen.1006516 (PMC5226683; doi:10.1371/journal.pgen.1006516)
Supplement: S5 Table — (PDF) [file pgen.1006516.s005.pdf]

Table S5. Results of qRT-PCR. The average Ct value per cell line per gene is shown for each of two experiments.

Experiment 1

	GUSB	*ELMO1*	*GPR141*	*NME8*	*SFRP4*	*EPDR1*	*STARD3NL*
WT 1	27.414	27.830	33.391	38.021	24.041	27.648	26.356
WT 2	27.649	25.908	33.867	Undetermined	28.011	28.657	25.932
WT 3	27.536	25.985	34.121	35.013	27.309	28.104	25.836
CFC 2	27.475	26.198	31.917	36.757	28.621	28.746	25.406
CFC 3	27.421	27.426	35.795	Undetermined	29.461	29.453	25.593
CFC 4	28.002	25.598	36.339	37.819	30.311	29.008	25.399

Experiment 2

	GUSB	*ELMO1*	*GPR141*	*NME8*	*SFRP4*	*EPDR1*	*STARD3NL*
WT 2	27.996	26.465	29.059	35.550	30.309	29.793	25.007
WT 3	27.965	25.879	28.691	32.287	30.411	28.184	24.595
CFC 1	27.281	25.319	29.557	37.721	27.993	27.607	24.398
CFC 3	26.876	25.078	26.215	36.848	31.822	31.202	24.049
CFC 4	26.595	26.186	28.422	35.222	28.926	26.166	22.248
