# Supplementary material for: Reverse Pathway Genetic Approach Identifies Epistasis in Autism Spectrum Disorders
Source: PLoS Genet. 2017 Jan 11;13(1):e1006516. doi: 10.1371/journal.pgen.1006516 (PMC5226683; doi:10.1371/journal.pgen.1006516)
Supplement: S6 Table — (PDF) [file pgen.1006516.s006.pdf]

Table S6. ASD datasets. The table describes the following information about each dataset used in the analysis: the final number of complete trio sets (unaffected mother and father, child with an ASD) in the homogeneous cluster, the genotyping array, and original publication of data.

Dataset	Number of Trios	Genotyping Platform	Publication
Autism Genetic Resource Exchange (AGRE)-Wang	535	Illumina HumanHap550 BeadChip	Wang, K. *et al*. (2009)(15)
AGRE-Weiss	194	Affymetrix 5.0 SNP array	Weiss, L. A. *et al*. (2009)(16)
Autism Genome Project (AGP)	1,936	Illumina Infinium 1Mv1 array	Anney, R. *et al.* (2010)(17)
Simons Simplex Collection (SSC)	1,414	Illumina Infinium 1Mv1 array, Illumina Infinium 1Mv3 (duo) array, and Illumina HumanOmni2.5M array	Chaste, P. *et al.* (2015)(18)
UCSF-Weiss	30	Affymetrix Axiom EUR arrays	Mitra, I. *et al*.
