# Supplementary material for: Reverse Pathway Genetic Approach Identifies Epistasis in Autism Spectrum Disorders
Source: PLoS Genet. 2017 Jan 11;13(1):e1006516. doi: 10.1371/journal.pgen.1006516 (PMC5226683; doi:10.1371/journal.pgen.1006516)
Supplement: S7 Table — (PDF) [file pgen.1006516.s007.pdf]

Table S7. Association signal at varying FDR thresholds. Percentage and number of Ras/MAPK polymorphisms meeting varying FDR thresholds for ASD association.

	**FDR 0.1**	**FDR 0.2**	**FDR 0.3**	**FDR 0.5**	**FDR 0.7**	**FDR 0.9**
**N SNPs**	0	228	263	323	1,179	1,736
**% above FDR**	0	9	10	13	47	69

Abbreviations: % above FDR: percentage of SNPs above FDR threshold; FDR: False Discovery Rate
