# Supplementary material for: Reverse Pathway Genetic Approach Identifies Epistasis in Autism Spectrum Disorders
Source: PLoS Genet. 2017 Jan 11;13(1):e1006516. doi: 10.1371/journal.pgen.1006516 (PMC5226683; doi:10.1371/journal.pgen.1006516)
Supplement: S8 Table — (PDF) [file pgen.1006516.s008.pdf]

Table S8. Table of Taqman probes. The catalog number by Taqman for each probe used by gene is listed in the table.

Gene name	Catalog (Taqman probes)
ELMO1	Hs00404979_m1
EPDR1	Hs01556067_m1
GPR141	Hs00704255_s1
GUSB	Hs00939627_m1
NME8	Hs00213301_m1
SFRP4	Hs00180066_m1
STARD3NL	Hs01106499_m1
