# Supplementary material for: Reverse Pathway Genetic Approach Identifies Epistasis in Autism Spectrum Disorders
Source: PLoS Genet. 2017 Jan 11;13(1):e1006516. doi: 10.1371/journal.pgen.1006516 (PMC5226683; doi:10.1371/journal.pgen.1006516)
Supplement: S1 Fig — (PDF) [file pgen.1006516.s009.pdf]

Figure S1: Most significant epistasis results in ASD. The graphs (A-F) display the regions containing the six most significant independent SNPs showing epistasis with Ras/MAPK SNPs. SNP position information is based on hg19 reference version and LD and recombination rate data are based on 1000 Genomes November 2014 EUR population. SNPs are colored based on linkage disequilibrium (LD) correlation (r^2), or colored gray if no LD information exists. The overlaid blue line corresponds to the recombination rate. Plot were created using LocusZoom(14) (see web resources).

A

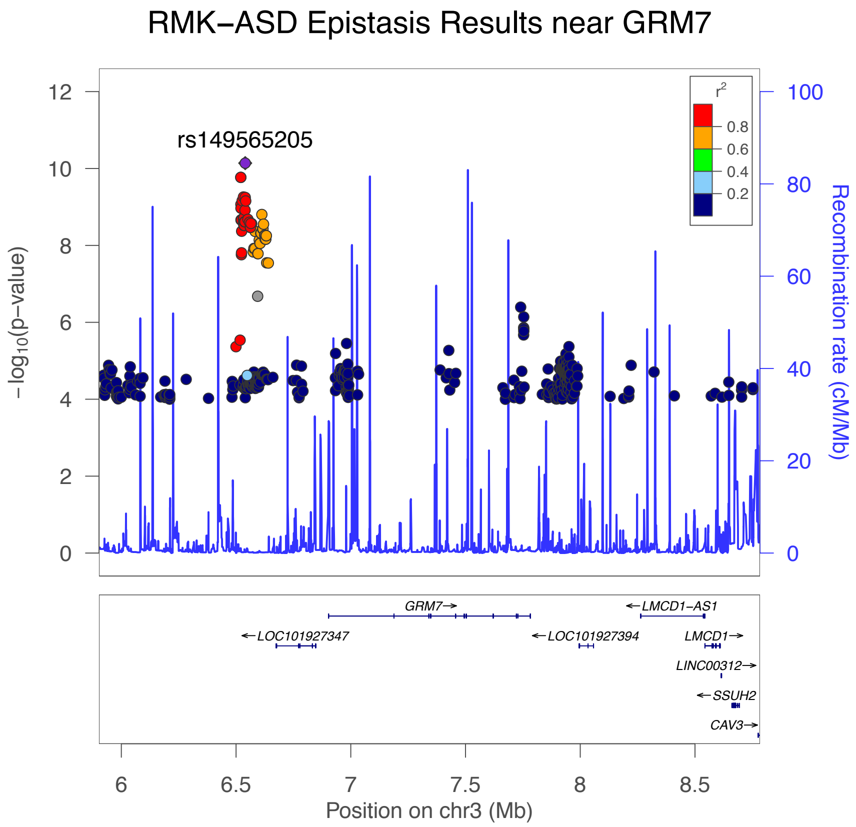

B

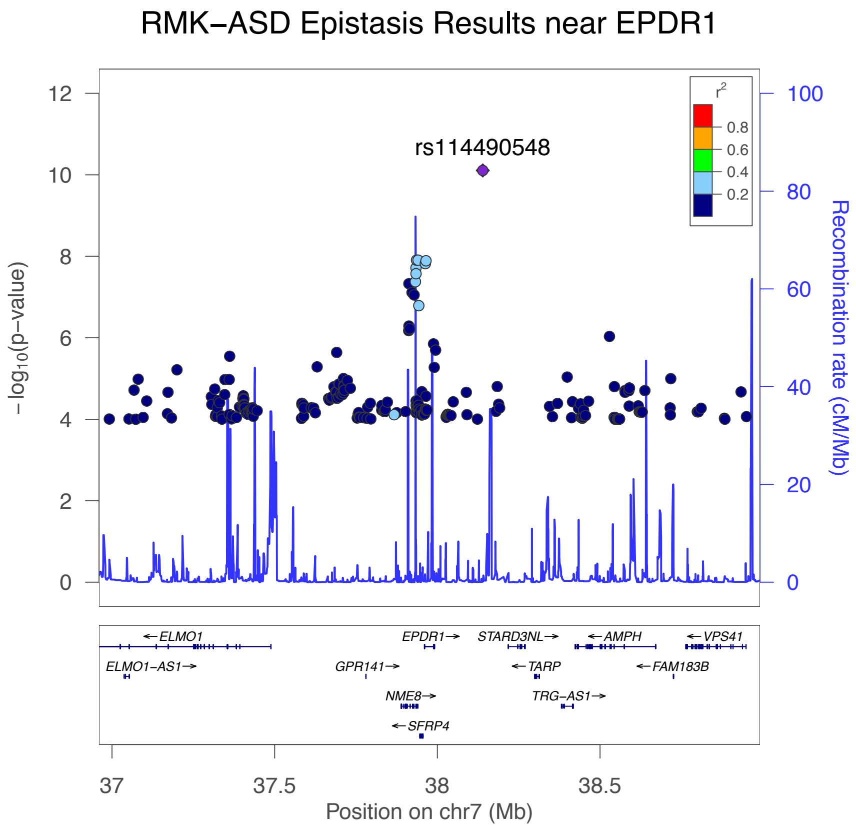

RMK−ASD Epistasis Results near EPDR1
rs114490548
r²
0.8
0.6
0.4
0.2
−log10(p−value)
Recombination rate (cM/Mb)
0
2
4
6
8
10
12
0
20
40
60
80
100
←ELMO1
ELMO1−AS1→
EPDR1→
GPR141→
NME8→
←SFRP4
STARD3NL→
←TARP
TRG−AS1→
←AMPH
←FAM183B
←VPS41
37
37.5
38
38.5
Position on chr7 (Mb)

C

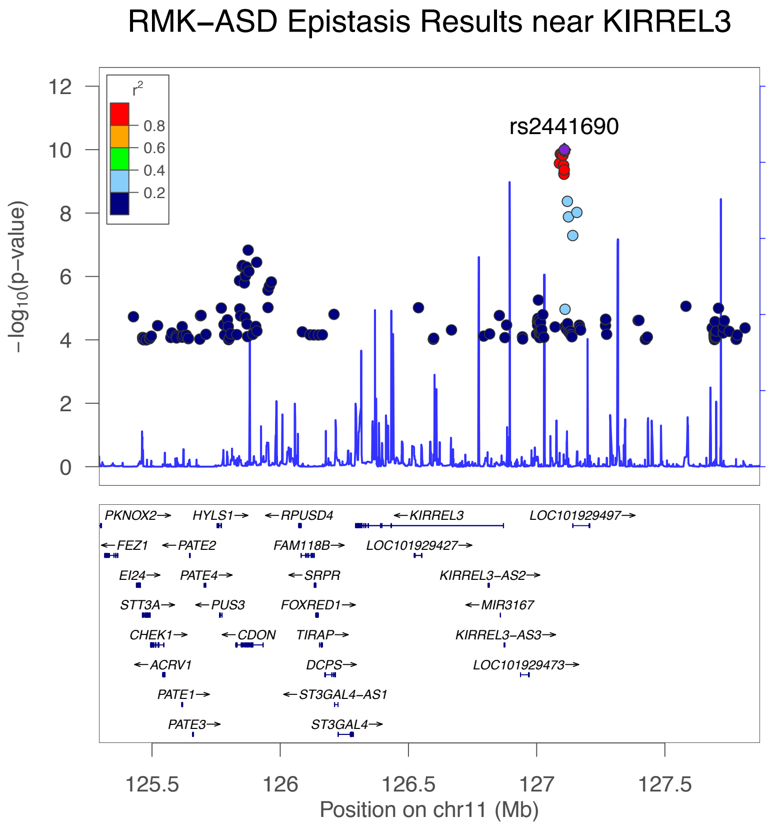

RMK−ASD Epistasis Results near KIRREL3
rs2441690
r²
0.8
0.6
0.4
0.2
−log10(p−value)
12
10
8
6
4
2
0
PKNOX2→
HYLS1→
←RPUSD4
←KIRREL3
LOC101929497→
←FEZ1
←PATE2
FAM118B→
LOC101929427→
EI24→
PATE4→
←SRPR
KIRREL3−AS2→
STT3A→
←PUS3
FOXRED1→
←MIR3167
CHEK1→
←CDON
TIRAP→
KIRREL3−AS3→
←ACRV1
DCPS→
LOC101929473→
PATE1→
←ST3GAL4−AS1
PATE3→
ST3GAL4→
125.5
126
126.5
127
127.5
Position on chr11 (Mb)

D

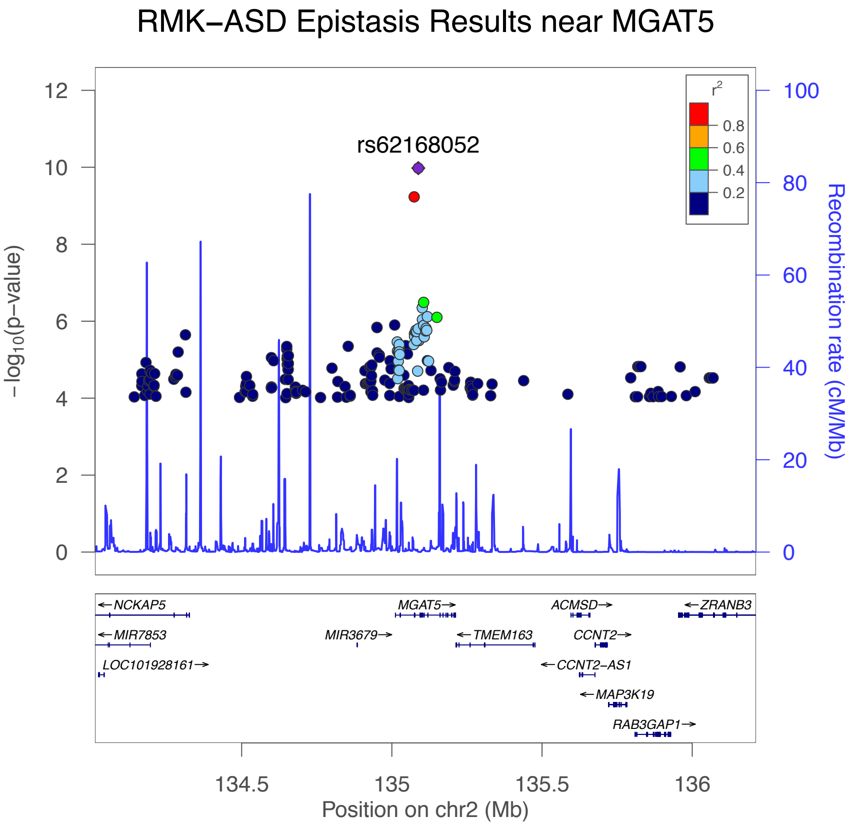

RMK−ASD Epistasis Results near MGAT5
rs62168052
−log10(p−value)
Recombination rate (cM/Mb)
r2
0.8
0.6
0.4
0.2
0
2
4
6
8
10
12
20
40
60
80
100
NCKAP5
MIR7853
LOC101928161
MIR3679
MGAT5
TMEM163
ACMSD
CCNT2
CCNT2−AS1
MAP3K19
RAB3GAP1
ZRANB3
134.5
135
135.5
136
Position on chr2 (Mb)

E

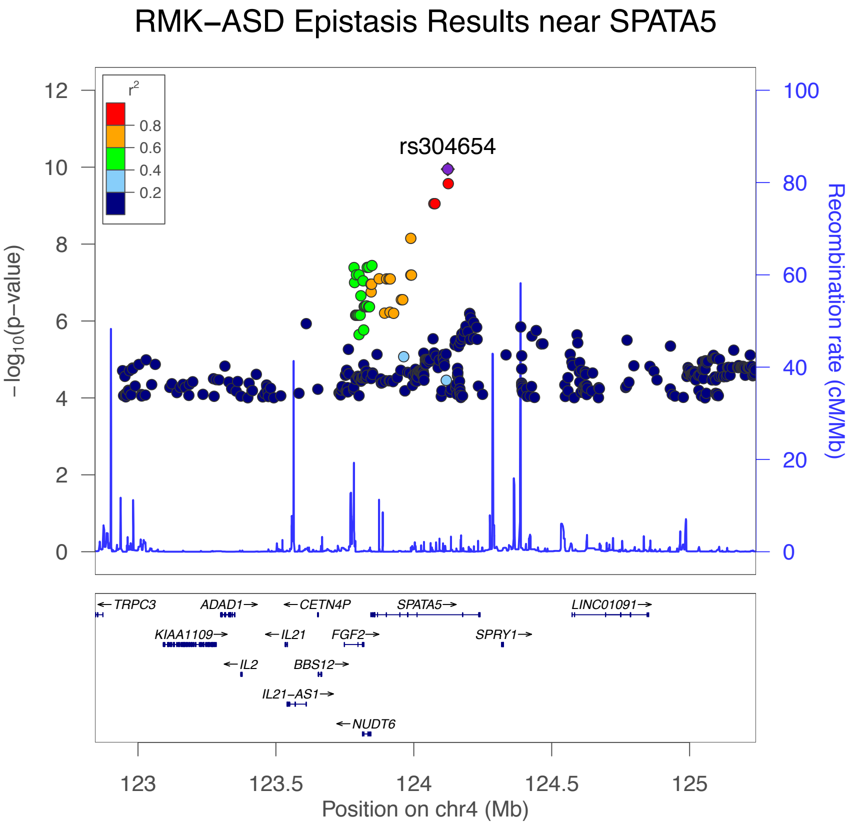
RMK−ASD Epistasis Results near SPATA5
r2
0.8
0.6
0.4
0.2
rs304654
−log10(p−value)
Recombination rate (cM/Mb)
0
2
4
6
8
10
12
0
20
40
60
80
100
TRPC3
ADAD1
CETN4P
SPATA5
LINC01091
KIAA1109
IL21
FGF2
SPRY1
IL2
BBS12
IL21−AS1
NUDT6
123
123.5
124
124.5
125
Position on chr4 (Mb)

F

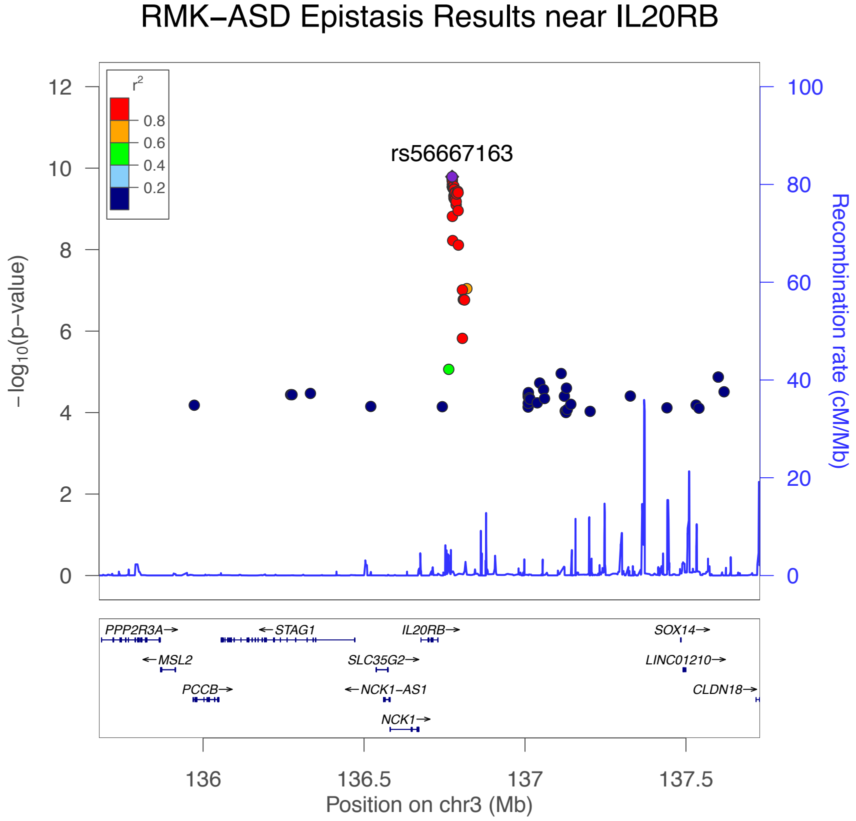
RMK−ASD Epistasis Results near IL20RB
r2
0.8
0.6
0.4
0.2
rs56667163
−log10(p−value)
Recombination rate (cM/Mb)
12
10
8
6
4
2
0
100
80
60
40
20
0
PPP2R3A
STAG1
IL20RB
SOX14
MSL2
SLC35G2
LINC01210
PCCB
NCK1−AS1
CLDN18
NCK1
136
136.5
137
137.5
Position on chr3 (Mb)
