# Supplementary material for: Reverse Pathway Genetic Approach Identifies Epistasis in Autism Spectrum Disorders
Source: PLoS Genet. 2017 Jan 11;13(1):e1006516. doi: 10.1371/journal.pgen.1006516 (PMC5226683; doi:10.1371/journal.pgen.1006516)

**Figure S3. Epistasis in sibling-paired SSC dataset.** The graph displays the number of epistasis tests (y-axis) in the ASD cases (dark gray, circle) and unaffected matched siblings (light gray, triangle) with *P*-value below thresholds (x-axis, left to right): $P < 2.9\text{x}10^{-9}$, $< 1.0\text{x}10^{-8}$, $P < 1.0\text{x}10^{-7}$, $P < 1.0\text{x}10^{-6}$, $P < 1.0\text{x}10^{-5}$, and $P < 1.0\text{x}10^{-4}$. The 2x2 chi-square test odds ratio (OR) is written for the epistasis results meeting nominal significance ($P < 10^{-6}$).

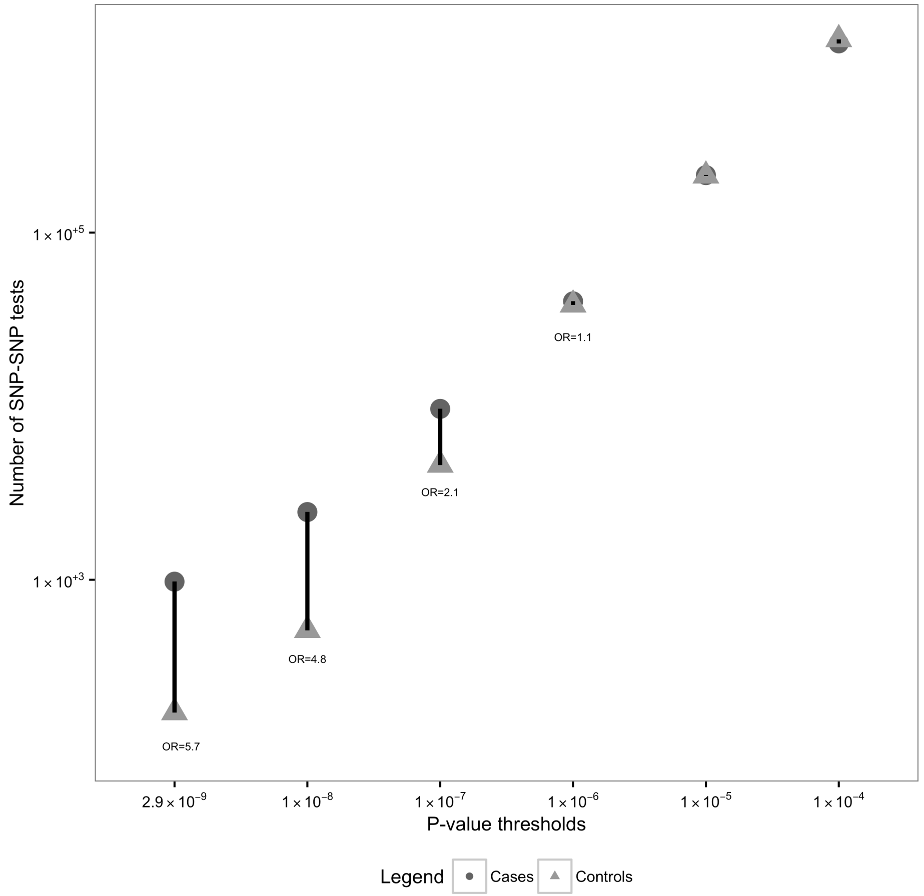

Supplement: S3 Fig — (PDF) [file pgen.1006516.s011.pdf]
