# Supplementary material for: Reverse Pathway Genetic Approach Identifies Epistasis in Autism Spectrum Disorders
Source: PLoS Genet. 2017 Jan 11;13(1):e1006516. doi: 10.1371/journal.pgen.1006516 (PMC5226683; doi:10.1371/journal.pgen.1006516)

Figure S4. Correlation plot of top Ras/MAPK - ASD epistasis results. The plot shows the -log10 *P*-values from the trio correlation test (y-axis) analysis and PLINK(63, 64) epistasis test (x-axis) in ASD cases for the most significant PLINK(63, 64) epistasis test results ($P < 1.0x10^{-6}$). The gray line graphs y=x.

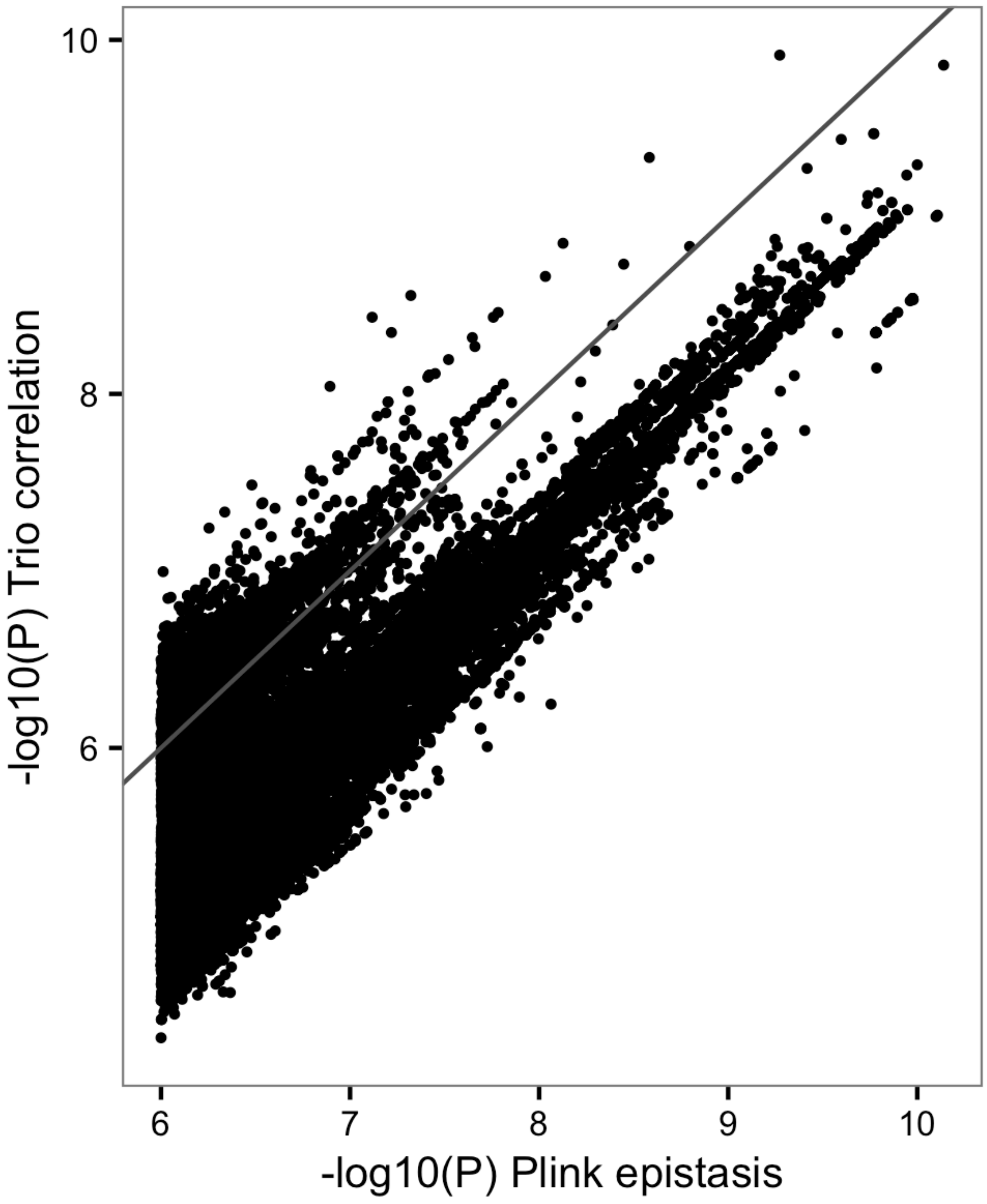

Supplement: S4 Fig — (PDF) [file pgen.1006516.s012.pdf]
