# Supplementary material for: Reverse Pathway Genetic Approach Identifies Epistasis in Autism Spectrum Disorders
Source: PLoS Genet. 2017 Jan 11;13(1):e1006516. doi: 10.1371/journal.pgen.1006516 (PMC5226683; doi:10.1371/journal.pgen.1006516)

Figure S5: Distribution of social responsiveness scores per RASopathy. The histograms display the frequency distribution (y-axis) of scaled social responsiveness scores (SRS) (x-axis) per RASopathy group. For each group, the mean of the SRS scores is 0 and variance is 1. The RASopathies group includes CFC, CS, NF1 and NS.

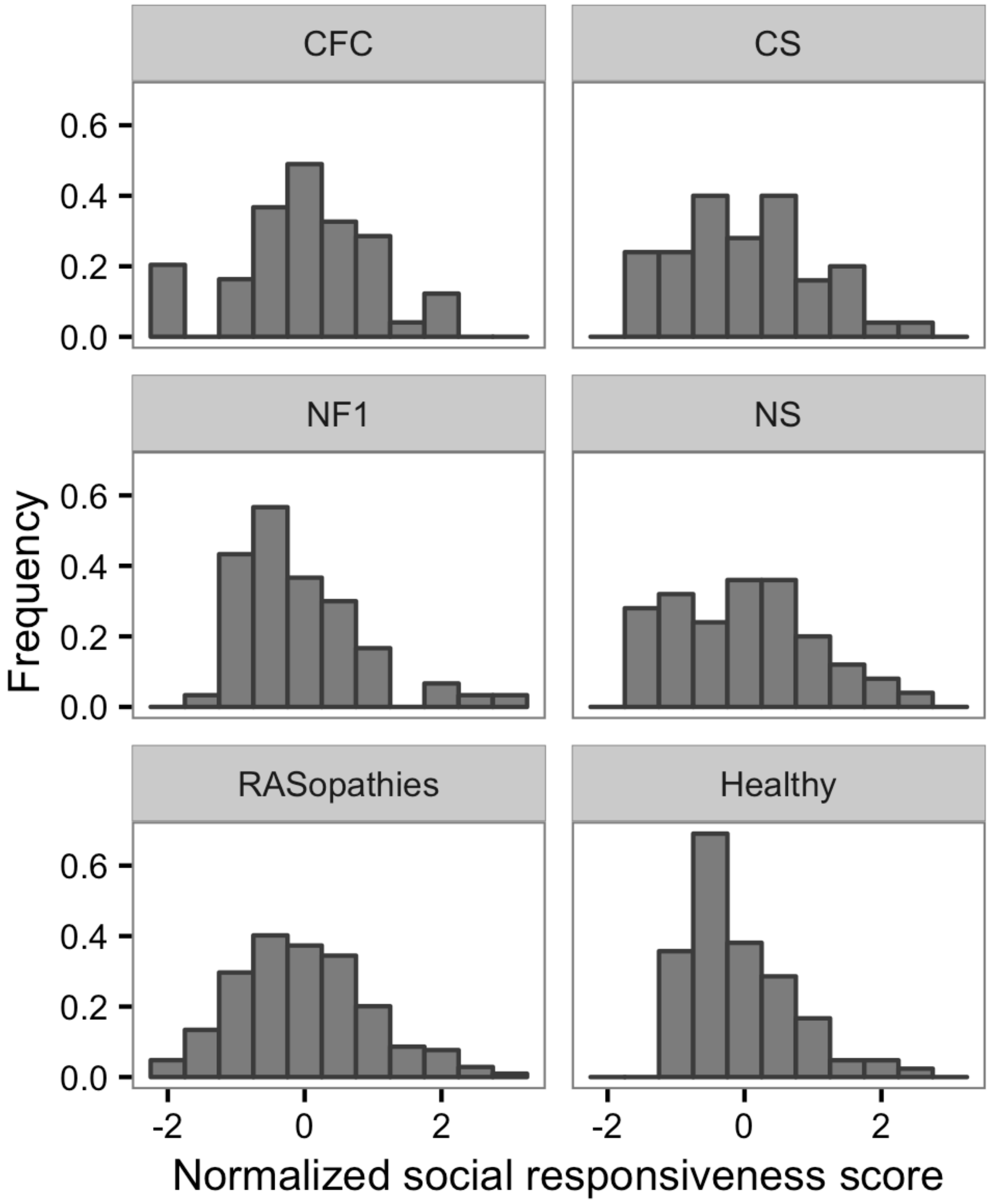

Supplement: S5 Fig — (PDF) [file pgen.1006516.s013.pdf]
