# Supplementary material for: Reverse Pathway Genetic Approach Identifies Epistasis in Autism Spectrum Disorders
Source: PLoS Genet. 2017 Jan 11;13(1):e1006516. doi: 10.1371/journal.pgen.1006516 (PMC5226683; doi:10.1371/journal.pgen.1006516)
Supplement: S6 Fig — (PDF) [file pgen.1006516.s014.pdf]

Figure S6. Distribution of social responsiveness scores for top six candidate modifiers. The asterisks on the RASopathies normalized social responsiveness score (SRS) distribution show the normalized SRS for heterozygous individuals for the top SNPs (MAF<=0.05) rs62621010 **(A)**, rs117802216 **(B)** and rs149068014 **(C)**. No homozygous individuals for the minor allele are found. Each individual is labeled by RASopathy. For the common SNPs (MAF>0.05) rs531418 **(D)**, rs11013152 **(E)** and rs1028948 **(F)**, the normalized social responsiveness score are shown by genotype in the boxplots. When heterogeneity is significant (see Table 2), only individuals in RASopathy groups that contribute to the meta-analysis evidence are shown.

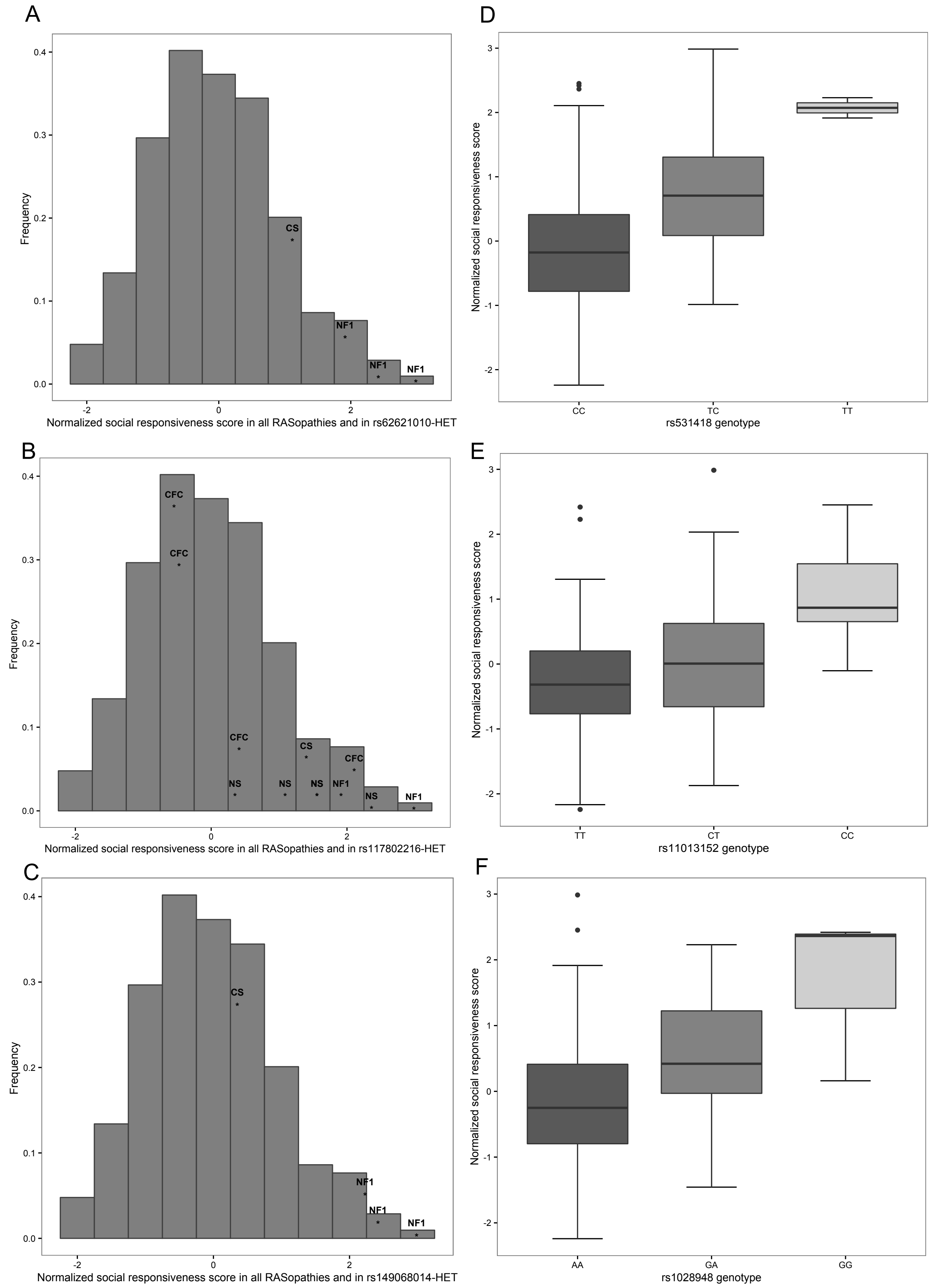

A
Frequency
CS
NF1
NF1
NF1
Normalized social responsiveness score in all RASopathies and in rs62621010-HET
B
Frequency
CFC
CFC
CFC
CS
CFC
NS
NS
NS
NF1
NS
NF1
Normalized social responsiveness score in all RASopathies and in rs117802216-HET
C
Frequency
CS
NF1
NF1
NF1
Normalized social responsiveness score in all RASopathies and in rs149068014-HET
D
Normalized social responsiveness score
CC
TC
TT
rs531418 genotype
E
Normalized social responsiveness score
TT
CT
CC
rs11013152 genotype
F
Normalized social responsiveness score
AA
GA
GG
rs1028948 genotype
