# Supplementary material for: Reverse Pathway Genetic Approach Identifies Epistasis in Autism Spectrum Disorders
Source: PLoS Genet. 2017 Jan 11;13(1):e1006516. doi: 10.1371/journal.pgen.1006516 (PMC5226683; doi:10.1371/journal.pgen.1006516)

Figure S7: Neural cell lines. CFC and control neural cultures derived from iPSC.(A) Electropherogram showing the heterozygote point mutation found in CFC patient iPSC lines (right) and the wild-type genotype from Control lines (left). (B) Neural culture differentiation from iPSC protocol outline based on Zhang *et al* (132). (C) Immunostaining of TUJ1, GFAP, MAP2, FOXP2, and CUX1 of CFC lines and control lines at the same time point (day 60) the qRT-PCR experiments were performed.

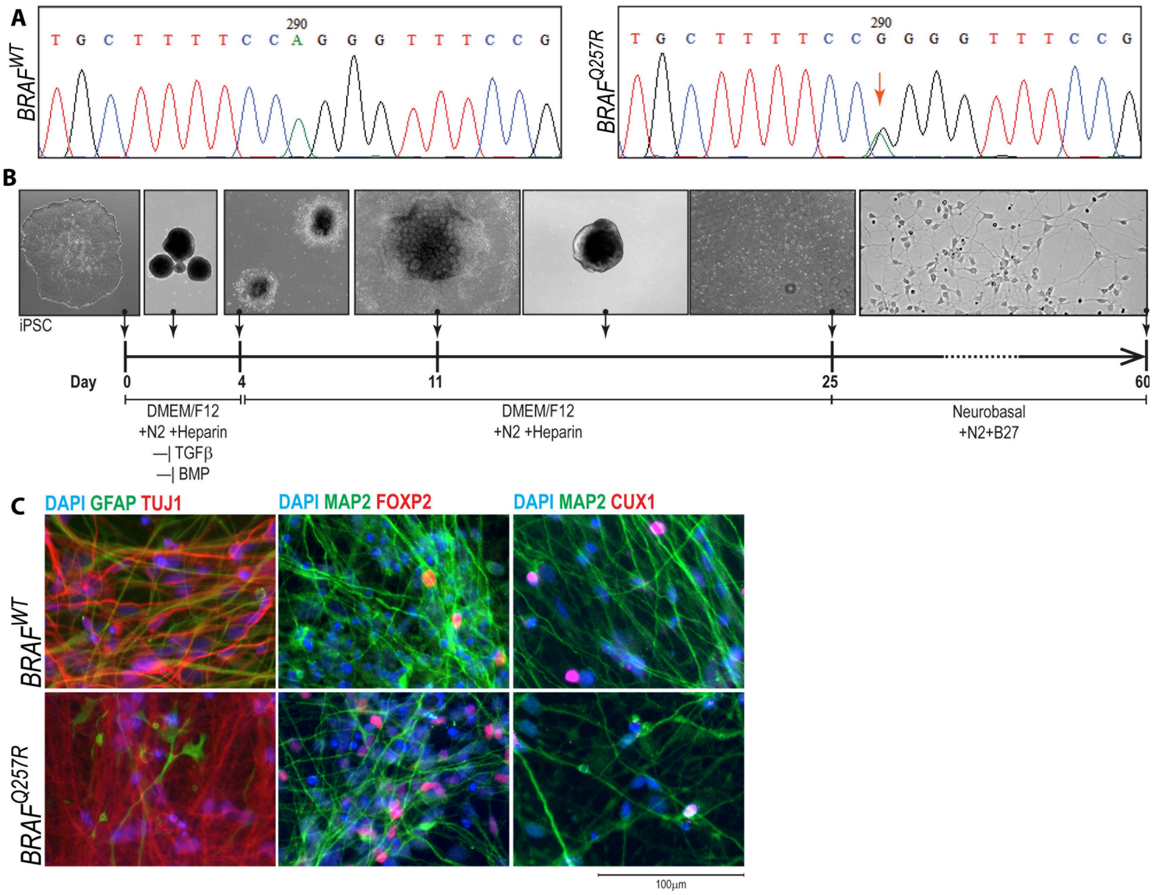

Supplement: S7 Fig — (PDF) [file pgen.1006516.s015.pdf]
