# Supplementary material for: Reverse Pathway Genetic Approach Identifies Epistasis in Autism Spectrum Disorders
Source: PLoS Genet. 2017 Jan 11;13(1):e1006516. doi: 10.1371/journal.pgen.1006516 (PMC5226683; doi:10.1371/journal.pgen.1006516)

Figure S9. QQ-plot for ASD association. The quantile-quantile plot is shown displaying observed vs. expected ASD association results for the TDT analysis of our trio dataset. Each point represents -log(P) for a SNP. The X-axis shows expected –log(P) and Y-axis shows observed –log(P), with the red line representing the null expection with a grey shaded 95% confidence interval. Different FDR thresholds are shown with dashed lines.

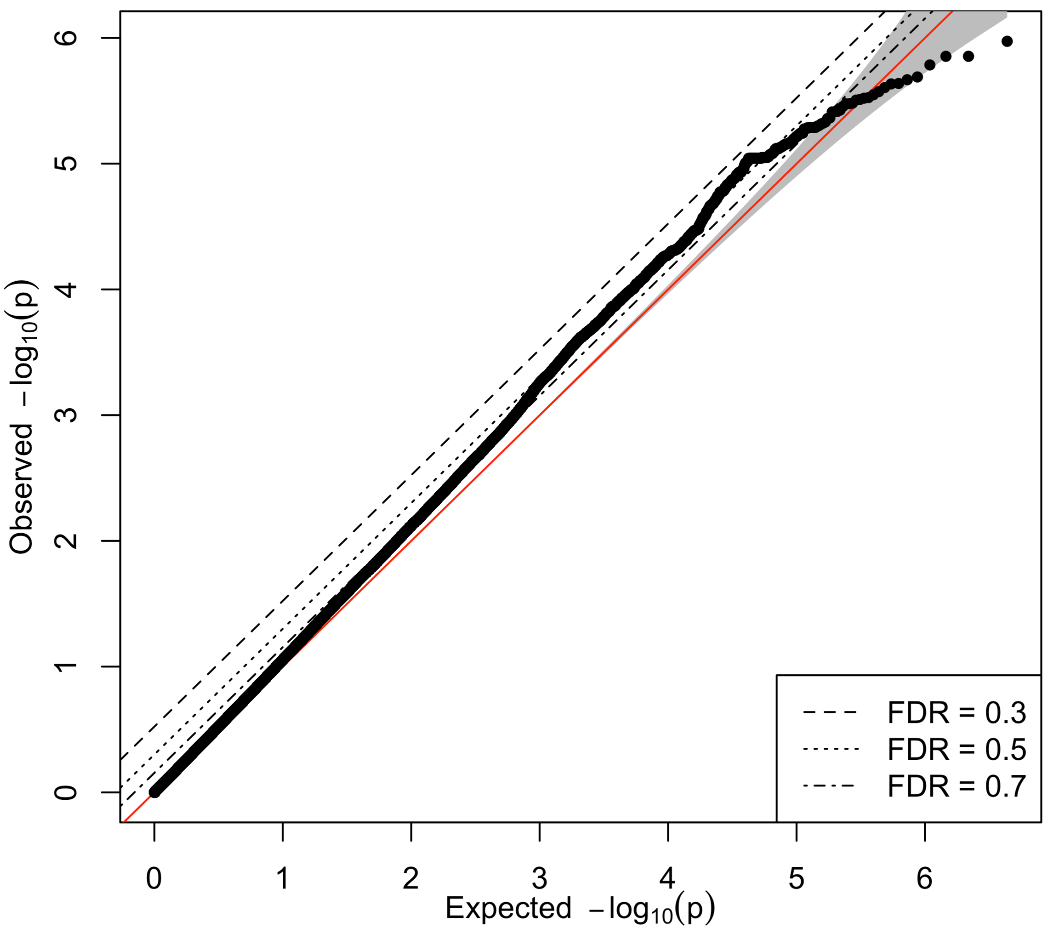

Supplement: S9 Fig — (PDF) [file pgen.1006516.s017.pdf]
